# Differential protection against SARS-CoV-2 reinfection pre- and post-Omicron

**DOI:** 10.1038/s41586-024-08511-9

**Published:** 2025-02-05

**Authors:** Hiam Chemaitelly, Houssein H. Ayoub, Peter Coyle, Patrick Tang, Mohammad R. Hasan, Hadi M. Yassine, Asmaa A. Al Thani, Zaina Al-Kanaani, Einas Al-Kuwari, Andrew Jeremijenko, Anvar Hassan Kaleeckal, Ali Nizar Latif, Riyazuddin Mohammad Shaik, Hanan F. Abdul-Rahim, Gheyath K. Nasrallah, Mohamed Ghaith Al-Kuwari, Adeel A. Butt, Hamad Eid Al-Romaihi, Mohamed H. Al-Thani, Abdullatif Al-Khal, Roberto Bertollini, Laith J. Abu-Raddad

**Affiliations:** 1 Infectious Disease Epidemiology Group, Weill Cornell Medicine–Qatar, Cornell University, Qatar Foundation – Education City, Doha, Qatar; 2 World Health Organization Collaborating Centre for Disease Epidemiology Analytics on HIV/AIDS, Sexually Transmitted Infections, and Viral Hepatitis, Weill Cornell Medicine–Qatar, Cornell University, Qatar Foundation – Education City, Doha, Qatar; 3 Department of Population Health Sciences, Weill Cornell Medicine, Cornell University, New York, NY USA; 4 Mathematics Program, Department of Mathematics, Statistics and Physics, College of Arts and Sciences, Qatar University, Doha, Qatar; 5 Department of Biomedical Science, College of Health Sciences, QU Health, Qatar University, Doha, Qatar; 6 Hamad Medical Corporation, Doha, Qatar; 7 Wellcome-Wolfson Institute for Experimental Medicine, Queens University, Belfast, UK; 8 Department of Pathology, Sidra Medicine, Doha, Qatar; 9 Department of Pathology and Molecular Medicine, McMaster University, Hamilton, Ontario Canada; 10 Biomedical Research Center, QU Health, Qatar University, Doha, Qatar; 11 Department of Public Health, College of Health Sciences, QU Health, Qatar University, Doha, Qatar; 12 Primary Health Care Corporation, Doha, Qatar; 13 Department of Medicine, Weill Cornell Medicine, Cornell University, New York, NY USA; 14 Ministry of Public Health, Doha, Qatar; 15 College of Health and Life Sciences, Hamad bin Khalifa University, Doha, Qatar

**Keywords:** Viral infection, Epidemiology

## Abstract

The severe acute respiratory syndrome coronavirus 2 (SARS-CoV-2) has rapidly evolved over short timescales, leading to the emergence of more transmissible variants such as Alpha and Delta^1–3^. The arrival of the Omicron variant marked a major shift, introducing numerous extra mutations in the spike gene compared with earlier variants^1,2^. These evolutionary changes have raised concerns regarding their potential impact on immune evasion, disease severity and the effectiveness of vaccines and treatments^1,3^. In this epidemiological study, we identified two distinct patterns in the protective effect of natural infection against reinfection in the Omicron versus pre-Omicron eras. Before Omicron, natural infection provided strong and durable protection against reinfection, with minimal waning over time. However, during the Omicron era, protection was robust only for those recently infected, declining rapidly over time and diminishing within a year. These results demonstrate that SARS-CoV-2 immune protection is shaped by a dynamic interaction between host immunity and viral evolution, leading to contrasting reinfection patterns before and after Omicron’s first wave. This shift in patterns suggests a change in evolutionary pressures, with intrinsic transmissibility driving adaptation pre-Omicron and immune escape becoming dominant post-Omicron, underscoring the need for periodic vaccine updates to sustain immunity.

## Abstract (web summary)

Two distinct patterns in the protective effect of natural infection against reinfection in the Omicron variant versus pre-Omicron eras show that SARS-CoV-2 immune protection is shaped by dynamic interaction between host immunity and viral evolution.

## Main

Contrary to an initial notion of slow evolution^1^, the severe acute respiratory syndrome coronavirus 2 (SARS-CoV-2) has demonstrated rapid evolutionary changes over short timescales^1–3^, consistent with those of coronaviruses and RNA viruses in general^1^. In the pre-Omicron era, various divergent lineages of SARS-CoV-2 emerged, giving rise to distinct variants such as Alpha, Beta and Delta^1^, each with its specific phenotypic traits^1,4–6^.

However, the emergence of the Omicron variant in late 2021 marked a major shift, as it harboured dozens of further mutations in the spike gene compared with its predecessors^1,2^. Since then, the Omicron lineage has continued to evolve, leading to the emergence of new variants^1,2^. These rapid evolutionary changes have sparked concerns regarding their potential implications for immune evasion, transmissibility, disease severity, diagnostic accuracy and the effectiveness of existing vaccines and treatments^1,3^.

SARS-CoV-2 infection has been shown to provide protective effects against reinfection under various scenarios, such as during the dominance of different strains^7–9^ and against specific variants^10–13^. Studies have also investigated the waning of immune protection conferred by pre-Omicron infection against both pre-Omicron and Omicron reinfections^7–9,14^. However, investigations into the durability of this protection have been limited with existing studies focusing on relatively short timeframes because of the recentness of the pandemic. Notably, the durability of Omicron infection in preventing reinfection with Omicron itself over extended periods of time remains unknown.

In this study, our objective was to examine the consequences of viral evolution, particularly the transition from the pre-Omicron era to the Omicron era, on the level and durability of protection provided by natural immunity. Natural immunity refers here to the protection gained from a previous infection against reinfection and against severe, critical or fatal coronavirus disease 2019 (COVID-19) on reinfection. We specifically investigated the level and durability of the effectiveness of Omicron infection in preventing reinfection with an Omicron virus, contrasting it with the effectiveness of a pre-Omicron infection in preventing reinfection with a pre-Omicron virus.

The effectiveness of natural infection against reinfection was estimated in Qatar’s population, both overall and by time since previous infection, using the test-negative, case-control study design^11,12,15,16^. Cases (SARS-CoV-2-positive tests) and controls (SARS-CoV-2-negative tests) were matched exactly one-to-two by sex, 10-year age group, nationality, number of coexisting conditions, number of vaccine doses, calendar week of the SARS-CoV-2 test, method of testing (polymerase chain reaction (PCR) or rapid antigen) and reason for testing, to balance observed confounders that could influence the risk of infection across the exposure groups^17^.

Whereas evidence on viral evolution and immunity at the molecular level indicates the continuing emergence of new SARS-CoV-2 variants with resistance to neutralization by plasma from recovered individuals and sera from vaccinated individuals^1,2,18–20^, the implications of these findings for the population-level phenomenon of immune protection remain uncertain. The present study provides direct population-level evidence contrasting the functional impact of viral evolution on immune protection in the pre-Omicron versus Omicron eras.

## Study populations

**Extended Data Fig. 1:**
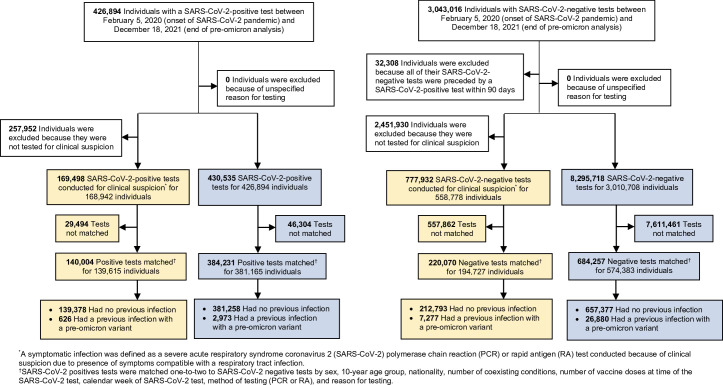
Study population selection process in the pre-omicron analysis. Flowchart describing the population selection process for investigating the effectiveness of a pre-omicron infection in preventing reinfection with a pre-omicron virus.

**Extended Data Fig. 2:**
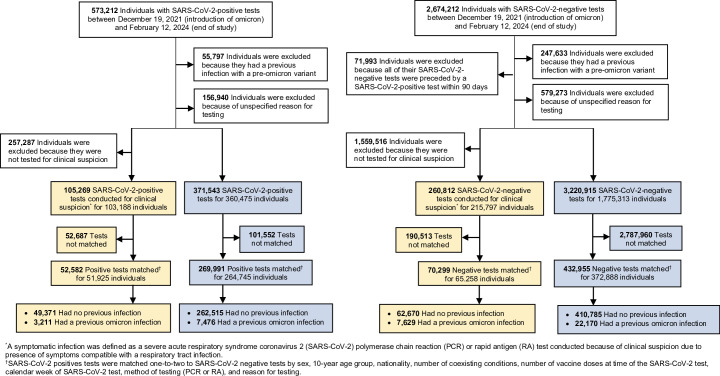
Study population selection process in the omicron analysis. Flowchart describing the population selection process for investigating the effectiveness of an omicron infection in preventing reinfection with an omicron virus.

**Extended Data Fig. 3:**
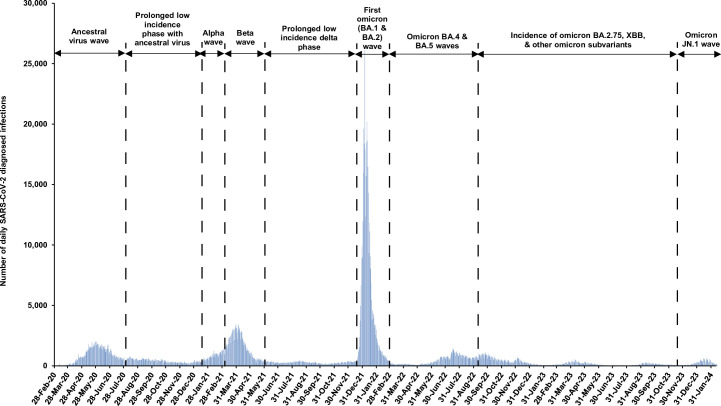
SARS-CoV-2 infection incidence in Qatar. Daily count of newly diagnosed SARS-CoV-2 infections up to the end of the study, between February 5, 2020 and February 12, 2024.

Extended Data Figures 1 and 2 illustrate the process of selecting the study populations for estimating the effectiveness of a pre-Omicron infection in preventing reinfection with a pre-Omicron virus and of an Omicron infection in preventing reinfection with an Omicron virus, respectively. Extended Data Figure 3 describes infection incidence at times of dominance of different SARS-CoV-2 variants in Qatar.

Extended Data Table 1 shows the characteristics of the study populations. The study was carried out on Qatar’s entire population; therefore, the study population is representative of the internationally diverse but predominantly young and male demographic of the country.

## Immune protection in the pre-Omicron era

**Fig. 1:**
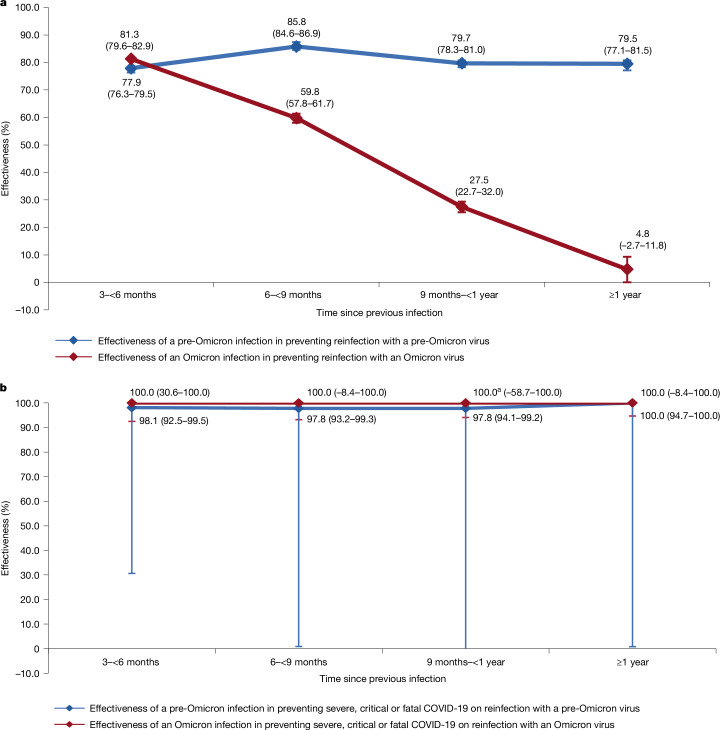
Effectiveness of previous infection against reinfection. **a**,**b**, Effectiveness of infection in preventing reinfection regardless of symptoms (**a**) and in preventing severe, critical or fatal COVID-19 on reinfection (**b**), by time since the previous infection. **a**, Includes 384,231 and 684,257 independent samples for cases and controls, respectively, in the pre-Omicron era analysis, and 269,991 and 432,955 independent samples for each of cases and controls, respectively, in the Omicron era analysis. Panel **b** includes 9,833 and 37,698 independent samples for cases and controls, respectively, in the pre-Omicron era analysis, and 230 and 650 independent samples for each of cases and controls, respectively, in the Omicron era analysis. Data are presented as effectiveness point estimates and corresponding 95% CIs. Error bars indicate the 95% CIs. Measures were not adjusted for multiplicity. In this figure, the 95% CIs are exceedingly small, rendering them barely noticeable for the protection against reinfection in both pre-Omicron and Omicron analyses, as well as for the protection against severe, critical or fatal COVID-19 on reinfection in the pre-Omicron era. This is attributed to the very large sample sizes in these analyses. However, because of the small number of cases of severe forms of COVID-19 in the Omicron era, the 95% CIs are very wide for the protection against severe, critical or fatal COVID-19 on reinfection in the Omicron era. ^a^The negative lower bound for the CI was truncated because the CI was too wide.

The overall effectiveness of a pre-Omicron infection in preventing reinfection, regardless of symptoms, with a pre-Omicron virus was estimated at 81.1% (95% confidence interval (CI), 80.4–81.8%) (Table 1a and Fig. 1a). The median duration between the previous infection and the study SARS-CoV-2 test was 252 days (interquartile range (IQR), 175–313 days). This robust protection showed limited waning over time after the previous infection. Effectiveness was 81.3% (95% CI, 80.6–82.1%) in the first year after the previous infection and 79.5% (95% CI, 77.1–81.5%) thereafter.

**Table 1:** Effectiveness of previous infection against reinfection

Effectiveness	Cases^a^		Controls^a^		Effectiveness^b^ (%) (95% CI)^c^	Cases^d^		Controls^d^		Effectiveness^b^ (%) (95% CI)^c^
	Previous infection (*n*)	No previous infection (*n*)	Previous infection (*n*)	No previous infection (*n*)		Previous infection (*n*)	No previous infection (*n*)	Previous infection (*n*)	No previous infection (*n*)	
**Effectiveness of a pre-Omicron infection in preventing**										
**(a) Reinfection with a pre-Omicron virus**						**(b) Severe, critical or fatal COVID-19 on reinfection with a pre-Omicron virus**				
Any previous infection	2,973	381,258	26,880	657,377	81.1 (80.4 to 81.8)	9	9,824	1,505	36,193	98.0 (96.1 to 99.0)
By time since previous infection										
Subgroup analysis 1										
3–<6 months	870	378,591	7,018	656,824	77.9 (76.3 to 79.5)	2	9,762	355	36,191	98.1 (92.5 to 99.5)
6–<9 months	686	378,980	8,330	656,893	85.8 (84.6 to 86.9)	3	9,771	463	36,190	97.8 (93.2 to 99.3)
9–<1 year	1,007	379,405	8,124	656,972	79.7 (78.3 to 81.0)	4	9,776	615	36,190	97.8 (94.1 to 99.2)
≥1 year	389	378,150	3,385	656,760	79.5 (77.1 to 81.5)	0	9,745	72	36,188	100.0 (94.7 to 100.0)^e^
Subgroup analysis 2										
<1 year	2,580	381,082	23,489	657,325	81.3 (80.6 to 82.1)	9	9,822	1,433	36,193	97.9 (95.9 to 98.9)
≥1 year	389	378,150	3,385	656,760	79.5 (77.1 to 81.5)	0	9,745	72	36,188	100.0 (94.7 to 100.0)^e^
**Effectiveness of an Omicron infection in preventing**										
**(c) Reinfection with an Omicron virus**						**(d) Severe, critical or fatal COVID-19 on reinfection with an Omicron virus**				
Any previous infection	7,476	262,515	22,170	410,785	53.6 (52.1 to 55.0)	0	230	20	630	100.0 (79.7 to 100.0)^e^
By time since previous infection										
Subgroup analysis 1										
3–<6 months	647	259,038	4,924	408,074	81.3 (79.6 to 82.9)	0	224	7	630	100.0 (30.6 to 100.0)^e^
6–<9 months	2,611	259,888	9,689	408,881	59.8 (57.8 to 61.7)	0	222	5	630	100.0 (−8.4 to 100.0)^e^
9–<1 year	1,916	258,775	4,095	408,566	27.5 (22.7 to 32.0)	0	223	3	630	100.0 (−58.7 to 100.0)^e^
≥1 year	1,981	258,550	2,989	408,594	4.8 (−2.7 to 11.8)	0	225	5	630	100.0 (−8.4 to 100.0)^e^
Subgroup analysis 2										
<1 year	5,318	261,806	18,950	409,948	59.5 (58.0 to 60.9)	0	227	15	630	100.0 (72.1 to 100.0)^e^
≥1 year	1,981	258,550	2,989	408,594	4.8 (−2.7 to 11.8)	0	225	5	630	100.0 (−8.4 to 100.0)^e^

^a^Cases (SARS-CoV-2-positive tests) and controls (SARS-CoV-2-negative tests) were matched exactly one-to-two by sex, 10-year age group, nationality, number of coexisting conditions, number of vaccine doses at time of the SARS-CoV-2 test, calendar week of the SARS-CoV-2 test, method of testing and reason for testing.

^b^Effectiveness of previous infection was estimated using the test-negative, case–control study design^15^.

^c^CIs were not adjusted for multiplicity and thus should not be used to infer definitive differences between different groups.

^d^Cases (SARS-CoV-2-positive tests) and controls (SARS-CoV-2-negative tests) were matched exactly one-to-five by sex, 10-year age group, nationality, number of coexisting conditions, number of vaccine doses at time of the SARS-CoV-2 test, calendar week of the SARS-CoV-2 test, method of testing and reason for testing. Severity, criticality and fatality were defined according to the World Health Organization guidelines.

^e^The 95% CI was estimated with the use of McNemar’s test because of zero events among exposed cases.

The effectiveness of a pre-Omicron infection in preventing symptomatic reinfection with a pre-Omicron virus demonstrated a similar pattern to that observed for any reinfection (Table 2a). The overall effectiveness against symptomatic reinfection was 86.8% (95% CI, 85.7–87.9%), with no evidence of waning over time after the previous infection. The median duration between the previous infection and the study SARS-CoV-2 test was 244 days (IQR, 169–303 days).

**Table 2:** Effectiveness of previous infection against symptomatic reinfection

Effectiveness	Cases^a^		Controls^a^		Effectiveness^b^ (%) (95% CI)^c^
	Previous infection (*n*)	No previous infection (*n*)	Previous infection (*n*)	No previous infection (*n*)	
**(a) Effectiveness of a pre-Omicron infection against symptomatic**^**d**^ **reinfection with a pre-Omicron virus**					
Any previous infection	626	139,378	7,277	212,793	86.8 (85.7 to 87.9)
By time since previous infection					
Subgroup analysis 1					
3–<6 months	177	137,936	2,027	212,561	85.2 (82.8 to 87.4)
6–<9 months	164	138,182	2,375	212,594	89.4 (87.5 to 91.0)
9 months–<1 year	236	138,453	2,116	212,662	84.6 (82.4 to 86.6)
≥1 year	44	137,707	753	212,536	90.0 (86.5 to 92.7)
Subgroup analysis 2					
<1 year	581	139,316	6,523	212,781	86.5 (85.3 to 87.6)
≥1 year	44	137,707	753	212,536	90.0 (86.5 to 92.7)
**(b) Effectiveness of an Omicron infection against symptomatic**^**d**^ **reinfection with an Omicron virus**					
Any previous infection	3,211	49,371	7,629	62,670	45.4 (42.5 to 48.2)
By time since previous infection					
Subgroup analysis 1					
3–<6 months	119	47,753	1,125	61,367	86.3 (83.2 to 88.9)
6–<9 months	606	48,046	2,236	61,610	64.0 (60.1 to 67.5)
9 months–<1 year	834	47,835	1,781	61,696	29.8 (22.6 to 36.4)
≥1 year	1,481	47,952	2,222	61,971	4.8 (−3.8 to 12.7)
Subgroup analysis 2					
<1 year	1,609	48,837	5,244	62,054	58.9 (56.1 to 61.5)
≥1 year	1,481	47,952	2,222	61,971	4.8 (−3.8 to 12.7)

^a^Cases (SARS-CoV-2-positive tests) and controls (SARS-CoV-2-negative tests) were matched exactly one-to-two by sex, 10-year age group, nationality, number of coexisting conditions, number of vaccine doses at time of the SARS-CoV-2 test, calendar week of the SARS-CoV-2 test, method of testing and reason for testing.

^b^Effectiveness of previous infection in preventing reinfection was estimated using the test-negative, case–control study design^15^.

^c^CIs were not adjusted for multiplicity and thus should not be used to infer definitive differences between different groups.

^d^A symptomatic infection was defined as a SARS-CoV-2 PCR or rapid antigen test conducted because of clinical suspicion due to the presence of symptoms compatible with a respiratory tract infection.

**Extended Data Fig. 4:**
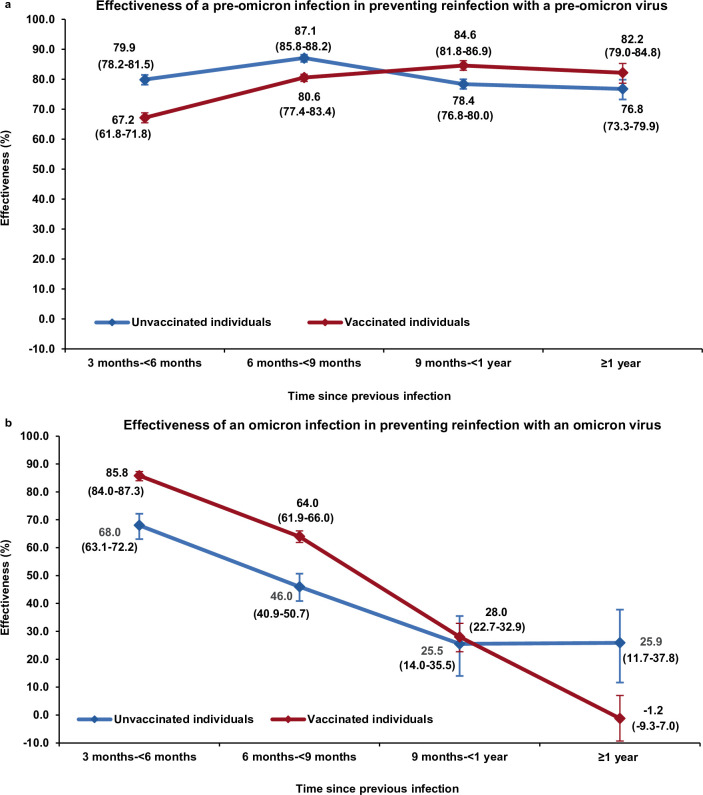
Effectiveness of previous infection in preventing reinfection by vaccination status. Effectiveness of infection in preventing reinfection by time since prior infection by vaccination status in the **a**) pre-omicron era and **b**) omicron era. **a** includes 353,485 and 627,836 independent samples for unvaccinated cases and controls, respectively, and 30,746 and 56,421 independent samples for vaccinated cases and controls, respectively. **b** includes 93,830 and 157,697 independent samples for unvaccinated cases and controls, respectively, and 176,161 and 275,258 independent samples for vaccinated cases and controls, respectively. Data are presented as effectiveness point estimates and corresponding 95% confidence intervals. Error bars indicate the 95% confidence intervals. Measures were not adjusted for multiplicity.

Subgroup analyses for both unvaccinated and vaccinated individuals yielded results similar to those of the main analysis (Table 3a and Extended Data Fig. 4a). The sensitivity analysis using a 40-day window^21^ for defining reinfection instead of the 90-day window also produced results consistent with the main analysis (Extended Data Table 2a).

**Table 3:** Effectiveness of previous infection against reinfection by vaccination status

Effectiveness	Cases^a^		Controls^a^		Effectiveness^b^ in (%) (95% CI)^c^
	Previous infection (*n*)	No previous infection (*n*)	Previous infection (*n*)	No previous infection (*n*)	
**(a) Effectiveness of a pre-Omicron infection against reinfection with a pre-Omicron virus**					
**Including only unvaccinated individuals**					
Any previous infection	2,247	351,238	20,768	607,068	81.5 (80.6 to 82.3)
By time since previous infection					
Subgroup analysis 1					
3–<6 months	665	349,165	5,938	606,661	79.9 (78.2 to 81.5)
6–<9 months	501	349,444	6,679	606,712	87.1 (85.8 to 88.2)
9 months–<1 year	846	349,835	6,399	606,804	78.4 (76.8 to 80.0)
≥1 year	219	348,655	1,736	606,573	76.8 (73.3 to 79.9)
Subgroup analysis 2					
<1 year	2,025	351,163	19,029	607,049	81.9 (81.0 to 82.7)
≥1 year	219	348,655	1,736	606,573	76.8 (73.3 to 79.9)
**Including only vaccinated individuals**					
Any previous infection	726	30,020	6,112	50,309	79.9 (78.2 to 81.4)
By time since previous infection					
Subgroup analysis 1					
3–<6 months	205	29,426	1,080	50,163	67.2 (61.8 to 71.8)
6–<9 months	185	29,536	1,651	50,181	80.6 (77.4 to 83.4)
9 months–<1 year	161	29,570	1,725	50,168	84.6 (81.8 to 86.9)
≥1 year	170	29,495	1,649	50,187	82.2 (79.0 to 84.8)
Subgroup analysis 2					
<1 year	555	29,919	4,460	50,276	79.0 (77.0 to 80.8)
≥1 year	170	29,495	1,649	50,187	82.2 (79.0 to 84.8)
**(b) Effectiveness of an Omicron infection against reinfection with an Omicron virus**					
**Including only unvaccinated individuals**					
Any previous infection	1,686	92,144	4,899	152,798	47.1 (43.7 to 50.4)
By time since previous infection					
Subgroup analysis 1					
3–<6 months	266	91,534	1,290	152,302	68.0 (63.1 to 72.2)
6–<9 months	778	91,662	2,328	152,492	46.0 (40.9 to 50.7)
9 months–<1 year	331	91,418	708	152,298	25.5 (14.0 to 35.5)
≥1 year	265	91,376	513	152,277	25.9 (11.7 to 37.8)
Subgroup analysis 2					
<1 year	1,404	92,030	4,371	152,704	49.4 (45.9 to 52.7)
≥1 year	265	91,376	513	152,277	25.9 (11.7 to 37.8)
**Including only vaccinated individuals**					
Any previous infection	5,790	170,371	17,271	257,987	55.5 (53.9 to 57.1)
By time since previous infection					
Subgroup analysis 1					
3–<6 months	381	167,504	3,634	255,772	85.8 (84.0 to 87.3)
6–<9 months	1,833	168,226	7,361	256,389	64.0 (61.9 to 66.0)
9 months–<1 year	1,585	167,357	3,387	256,268	28.0 (22.7 to 32.9)
≥1 year	1,716	167,174	2,476	256,317	−1.2 (−9.3 to 7.0)
Subgroup analysis 2					
<1 year	3,914	169,776	14,579	257,244	62.5 (60.9 to 64.0)
≥1 year	1,716	167,174	2,476	256,317	−1.2 (−9.3 to 7.0)

^a^Cases (SARS-CoV-2-positive tests) and controls (SARS-CoV-2-negative tests) were matched exactly one-to-two by sex, 10-year age group, nationality, number of coexisting conditions, number of vaccine doses at time of the SARS-CoV-2 test, calendar week of the SARS-CoV-2 test, method of testing and reason for testing.

^b^Effectiveness of previous infection in preventing reinfection was estimated using the test-negative, case–control study design^15^.

^c^CIs were not adjusted for multiplicity and thus should not be used to infer definitive differences between different groups.

The overall effectiveness of a pre-Omicron infection in preventing severe, critical or fatal COVID-19 on reinfection with a pre-Omicron virus was 98.0% (95% CI, 96.1–99.0%), with no observed waning over time after the previous infection (Table 1b and Fig. 1b).

## Immune protection in the Omicron era

The overall effectiveness of an Omicron infection in preventing reinfection, regardless of symptoms, with an Omicron virus was estimated at 53.6% (95% CI, 52.1–55.0%) (Table 1c and Fig. 1a). The median duration between the previous infection and the study SARS-CoV-2 test was 245 days (IQR, 191–311 days).

This effectiveness demonstrated a rapid decline over time after the previous infection, decreasing from 81.3% (95% CI, 79.6–82.9%) within 3 to less than 6 months after the previous infection to 59.8% (95% CI, 57.8–61.7%) in the subsequent 3 months, and further dropping to 27.5% (95% CI, 22.7–32.0%) in the subsequent 3 months (Table 1c and Fig. 1a). Ultimately, it reached a negligible level after 1 year. The effectiveness was 59.5% (95% CI, 58.0–60.9%) in the first year after the previous infection and 4.8% (95% CI, −2.7–11.8%) thereafter.

The effectiveness of an Omicron infection in preventing symptomatic reinfection with an Omicron virus demonstrated a similar pattern to that observed for any reinfection (Table 2b). The overall effectiveness against symptomatic reinfection was 45.4% (95% CI, 42.5–48.2%), with a rapid decline observed over time after the previous infection. The median duration between the previous infection and the study SARS-CoV-2 test was 301 days (IQR, 225–457 days).

Subgroup analyses for both unvaccinated and vaccinated individuals yielded results similar to those of the main analysis (Table 3b and Extended Data Fig. 4b). The sensitivity analysis using a 40-day window^21^ for defining reinfection instead of the 90-day window also produced results consistent with the main analysis (Extended Data Table 2b).

The overall effectiveness of an Omicron infection in preventing severe, critical or fatal COVID-19 on reinfection with an Omicron virus was 100% (95% CI, 79.9–100%), with no cases of reinfection progressing to severe, critical or fatal COVID-19 (Table 1d). There was no evidence for a waning in this effectiveness over time after the previous infection (Fig. 1b).

## Further validation analyses

### Previous infection misclassification

**Extended Data Fig. 5:**
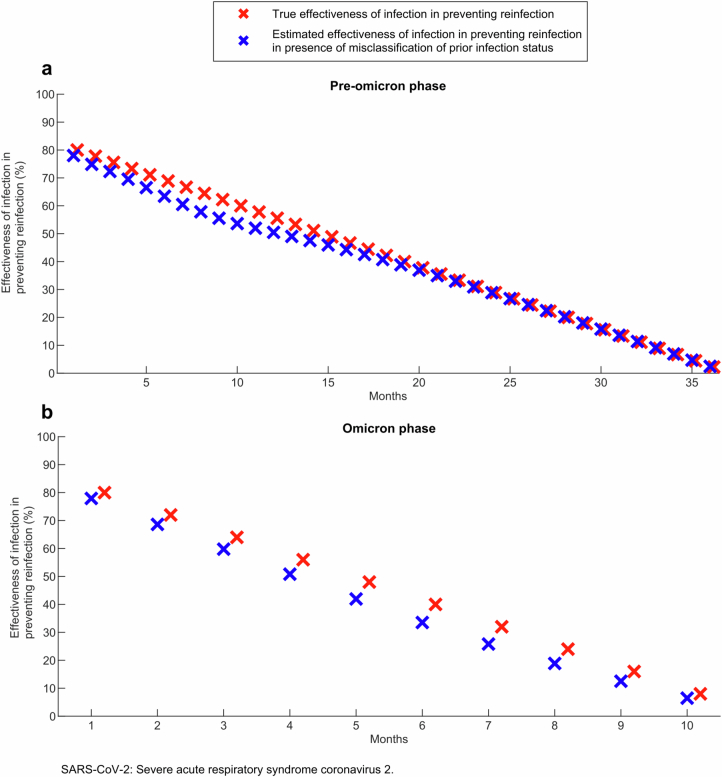
Impact of bias due to misclassification of prior infection status. Impact of bias due to misclassification of prior infection status in the test-negative design, assuming 90% of SARS-CoV-2 infections are undocumented. The figure shows the simulated estimated effectiveness of infection against reinfection in presence of misclassification of prior infection status compared to the true effectiveness of infection against reinfection in the pre-omicron **a**) and omicron **b**) phases of the pandemic.

Under-ascertainment of infection leads to misclassification of previous infection status in the test-negative design used in this study, potentially biasing the estimates^15^. Extended Data Figure 5 presents the results of mathematical modelling simulations that evaluated the impact of extreme under-ascertainment (90% of SARS-CoV-2 infections are undocumented) on the estimated waning pattern of immune protection during the pre-Omicron and Omicron eras.

The estimated effectiveness in presence of this bias, in both pre-Omicron and Omicron eras, was comparable to, but slightly lower than, the true effectiveness (Extended Data Fig. 5). This underestimation was larger in the Omicron analysis. However, in both analyses, this bias did not affect the estimated duration of immune protection, which is the central focus of our study.

### Coexisting conditions misclassification

Coexisting conditions were identified by analysing electronic health record encounters within the national public healthcare system’s database (Supplementary Methods section 1). However, this approach may not capture all conditions, as some may be undiagnosed or diagnosed at private facilities.

**Extended Data Fig. 6:**
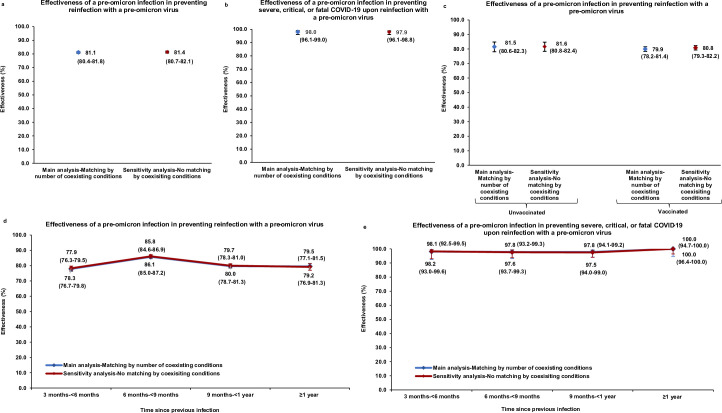
Impact of misclassification of coexisting condition status on the results for the pre-omicron era. The figure compares the main analysis results to those of a sensitivity analysis, where matching by the number of coexisting conditions was entirely removed to simulate complete under-ascertainment of coexisting conditions. The comparisons include: **a**) overall effectiveness of infection in preventing reinfection, **b**) overall effectiveness of infection in preventing severe, critical, or fatal COVID-19 upon reinfection, **c**) overall effectiveness of infection in preventing reinfection for each of vaccinated and unvaccinated individuals, **d**) effectiveness of infection in preventing reinfection by time since prior infection, and **e**) effectiveness of infection in preventing severe, critical, or fatal COVID-19 upon reinfection by time since prior infection. Main analysis includes 384,231 and 684,257 independent samples for cases and controls, respectively. Sensitivity analysis includes 401,686 and 723,529 independent samples for cases and controls, respectively. Data are presented as effectiveness point estimates and corresponding 95% confidence intervals. Error bars indicate the 95% confidence intervals. Measures were not adjusted for multiplicity.

**Extended Data Fig. 7:**
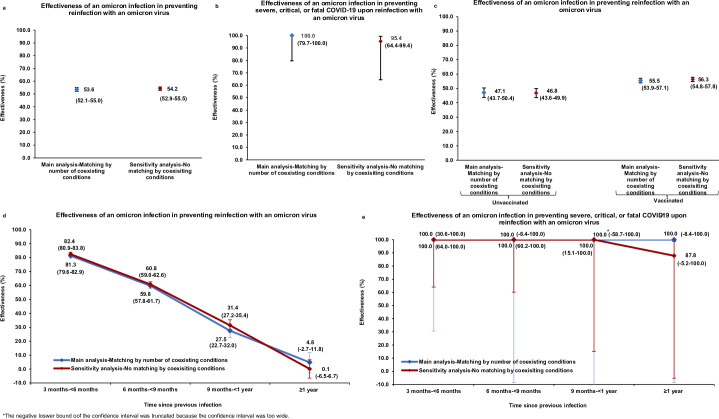
Impact of misclassification of coexisting condition status on the results for the omicron era. The figure compares the main analysis results to those of a sensitivity analysis, where matching by the number of coexisting conditions was entirely removed to simulate complete under-ascertainment of coexisting conditions. The comparisons include: **a**) overall effectiveness of infection in preventing reinfection, **b**) overall effectiveness of infection in preventing severe, critical, or fatal COVID-19 upon reinfection, **c**) overall effectiveness of infection in preventing reinfection for each of vaccinated and unvaccinated individuals, **d**) effectiveness of infection in preventing reinfection by time since prior infection, and **e**) effectiveness of infection in preventing severe, critical, or fatal COVID-19 upon reinfection by time since prior infection. Main analysis includes 269,991 and 432,955 independent samples for cases and controls, respectively. Sensitivity analysis includes 287,576 and 464,064 independent samples for cases and controls, respectively. Data are presented as effectiveness point estimates and corresponding 95% confidence intervals. Error bars indicate the 95% confidence intervals. Measures were not adjusted for multiplicity.

The sensitivity analysis, which removed matching by the number of coexisting conditions to simulate a scenario of complete under-ascertainment, showed results nearly identical to the main analysis across the various estimates (Extended Data Figs. 6 and 7). This consistency was observed for both the pre-Omicron and Omicron eras, including overall effectiveness against any infection and against infection in both unvaccinated and vaccinated individuals, as well as effectiveness against severe, critical or fatal COVID-19 on reinfection, and effectiveness over time after the previous infection.

### Validation using a cohort study design

This study was conducted using the test-negative design^15^. To validate the findings, two national, matched, retrospective cohort studies were also conducted: one for the pre-Omicron era and one for the Omicron era.

**Extended Data Fig. 8:**
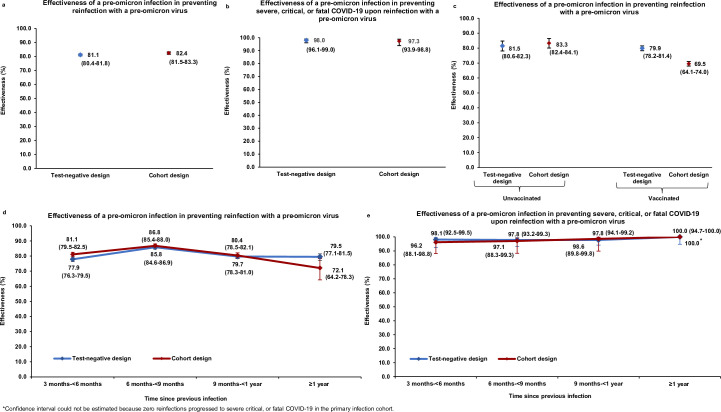
Validation of the results for the pre-omicron era using a cohort study design. The figure compares the main analysis results obtained using the test-negative design to the results obtained using the cohort study design. The comparisons include: **a**) overall effectiveness of infection in preventing reinfection, **b**) overall effectiveness of infection in preventing severe, critical, or fatal COVID-19 upon reinfection, **c**) overall effectiveness of infection in preventing reinfection for each of vaccinated and unvaccinated individuals, **d**) effectiveness of infection in preventing reinfection by time since prior infection, and **e**) effectiveness of infection in preventing severe, critical, or fatal COVID-19 upon reinfection by time since prior infection. Analysis using the test-negative design includes 384,231 and 684,257 independent samples for cases and controls, respectively. Analysis using the cohort design includes 339,019 independent samples for each of the primary infection and uninfected cohorts. Data are presented as effectiveness point estimates and corresponding 95% confidence intervals. Error bars indicate the 95% confidence intervals. Measures were not adjusted for multiplicity.

**Extended Data Fig. 9:**
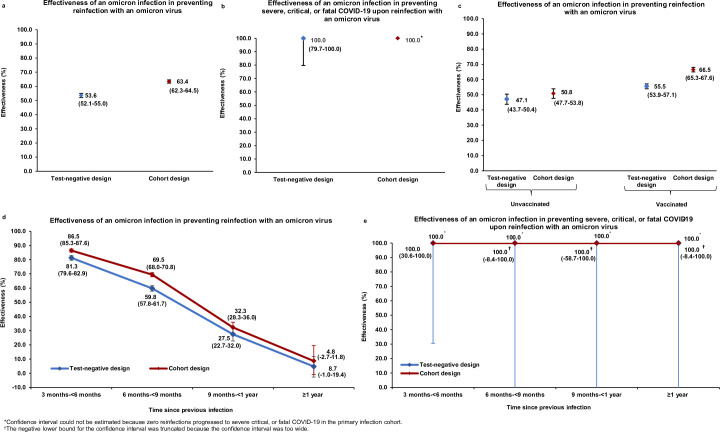
Validation of the results for the omicron era using a cohort study design. The figure compares the main analysis results obtained using the test-negative design to the results obtained using the cohort study design. The comparisons include: **a**) overall effectiveness of infection in preventing reinfection, **b**) overall effectiveness of infection in preventing severe, critical, or fatal COVID-19 upon reinfection, **c**) overall effectiveness of infection in preventing reinfection for each of vaccinated and unvaccinated individuals, **d**) effectiveness of infection in preventing reinfection by time since prior infection, and **e**) effectiveness of infection in preventing severe, critical, or fatal COVID-19 upon reinfection by time since prior infection. Analysis using the test-negative design includes 269,991 and 432,955 independent samples for cases and controls, respectively. Analysis using the cohort design includes 329,117 independent samples for each of the primary infection and uninfected cohorts. Data are presented as effectiveness point estimates and corresponding 95% confidence intervals. Error bars indicate the 95% confidence intervals. Measures were not adjusted for multiplicity.

The estimates from both the test-negative study design and the cohort study design were consistent across the various outcomes in both the pre-Omicron and Omicron eras (Extended Data Figs. 8 and 9). These outcomes included overall effectiveness against any infection and against infection in both unvaccinated and vaccinated individuals, as well as effectiveness against severe, critical or fatal COVID-19 on reinfection, and effectiveness over time after the previous infection. Whereas the test-negative design yielded lower estimates overall, particularly in the Omicron analysis, both study designs produced similar results for the duration of immune protection, validating the findings of the test-negative design.

## Discussion

The results show two distinct patterns in the protective effect of natural infection against reinfection in the Omicron era compared to the pre-Omicron era. Before the emergence of Omicron, natural infection offered robust protection against reinfection, with roughly 80% effectiveness and minimal signs of waning over time after the infection. However, during the Omicron era, this protection was strong only for recently infected individuals, rapidly declining over time after the infection and ultimately diminishing within a year. These patterns were consistent regardless of whether any infection or only symptomatic infection was considered as an outcome, and for both vaccinated and unvaccinated populations.

The two distinct patterns observed in the Omicron versus pre-Omicron eras provide population-level results that validate previous experimental molecular evidence^1,2,18–20^, and are probably the result of a complex interplay of several interrelated factors, in addition to waning immunity, immune evasion and the accelerated and convergent evolution of Omicron, such as immune imprinting, varying immunogenicity, global population immunity faced by the strains and population characteristics associated with infections at different stages of the pandemic.

Whereas these factors are interconnected and challenging to disentangle, the observed differences in protection against reinfection may stem from distinct evolutionary pressures acting on SARS-CoV-2 during the pre-Omicron and Omicron eras. In the pre-Omicron era, with a large proportion of individuals remaining immune naive because of non-pharmaceutical interventions and delayed scale-up of vaccination, intrinsic transmissibility may have been the primary driver of viral adaptation. This was evidenced by the emergence of more transmissible variants such as Alpha^4,22,23^ and Delta^24,25^. Conversely, following the very large and widespread Omicron wave in early 2022 (Extended Data Fig. 3)^26^, most individuals possessed some level of immunity, either from infection or vaccination. This may have shifted the dominant evolutionary pressure towards immune escape through not only antigenic drift, but also recombination and convergent evolution as the adaptive mechanisms for the virus^2,18,27,28^.

This shift in evolutionary pressure can explain the observed rapid decline in natural immunity protection against reinfection in the Omicron era and suggests an accelerated evolution of SARS-CoV-2 towards enhanced immune evasion in the current stage of the pandemic. The extent to which these findings describe an ecological survival strategy for novel respiratory viruses capable of rapid mutation, such as RNA viruses, as they transition from emergence to endemicity remains unknown, as does the relevance of these findings to other circulating respiratory viruses.

Immune imprinting, the memory recall behaviour of the immune system, in which the outcome is influenced by the antigenic distance between the ancestral strain and the variant^27,29–32^, may also help to explain these findings. This phenomenon, in which the specific sequence of immunological events can either enhance or compromise future immune responses to variant infections, may shape the diversity of polyclonal neutralizing antibodies elicited by Omicron breakthrough infections, potentially contributing to the observed patterns^27,29–33^.

An important finding is the robust and durable protection against severe COVID-19 on reinfection in both pre-Omicron and Omicron eras, with no observed waning in this protection. This distinct pattern suggests the involvement of different immune system components in protecting against non-severe versus severe reinfection. Whereas humoral immunity, mediated by neutralizing antibodies that block viral entry^34,35^, is the primary driver of protection against non-severe reinfection, it faces intense pressure from viral evolution towards immune escape. Conversely, protection against severe reinfection seems strongly influenced by cellular immunity through memory T cells^36–38^, which appears to be largely conserved^37,38^, perhaps indicating limited pressure on cellular immunity from the evolutionary forces driving immune escape.

Both vaccinated and unvaccinated populations showed similar contrasting patterns of protection between the pre-Omicron and Omicron eras (Extended Data Fig. 4). However, small quantitative differences in effectiveness were observed between these groups. These differences might be attributed to variations in the distribution of time intervals between the previous infection and the study test, a consequence of the changing dynamics of infection waves and vaccination scale-up over time. Previous studies suggest that immune imprinting effects might also have a role in explaining these differences^11,12,30^, but further investigation is needed to clarify these patterns.

Our findings at this stage of the pandemic corroborate earlier observations, demonstrating the consistently reduced severity of reinfections compared to primary infections^9,14,39^, even when both involve the Omicron variant. The results also confirm the strong protective effect of pre-Omicron immunity against pre-Omicron virus^4,7–10,40,41^. Furthermore, they highlight an association between viral immune evasion and accelerated waning of immune protection. This accelerated waning was previously observed in the rapid decline of pre-Omicron immunity, induced by both vaccination or natural infection, against the Omicron variant on its emergence^14,42–45^. Collectively, these findings suggest that immune evasion and subsequent rapid waning of immunity hinder the development of long-term population immunity to SARS-CoV-2, potentially leading to periodic waves of infection^46^, similar to those observed for common-cold coronaviruses^47,48^ and influenza^49,50^.

This study has limitations. The study is based on documented SARS-CoV-2 infections, but many infections may not have been documented, especially since the reduction in testing starting from 1 November 2022. However, this under-documentation of infection, a source of misclassification bias of previous infection status, may not have appreciably affected our findings based on our earlier analysis of the test-negative design methodology^15^. It should not have affected the estimated duration of immune protection, as demonstrated in the extra mathematical modelling analyses conducted in this study (Extended Data Fig. 5).

Depletion of the primary-infection cohort within the population owing to COVID-19 mortality at the time of primary infection can skew this cohort towards healthier individuals^51^. This skew could potentially lead to an overestimation of the effectiveness of infection in preventing severe, critical or fatal COVID-19 on reinfection. Nevertheless, given the low COVID-19 mortality rate in Qatar’s predominantly young population (less than 0.1% of primary infections)^26,52,53^, a survival effect is not likely to appreciably alter the observed high effectiveness of previous infection in mitigating the severity of reinfection.

In the sensitivity analysis using a 40-day time window to define reinfection, as opposed to the conventional 90-day time window^21^ (Extended Data Table 2), estimates for both overall protection and protection lasting less than 1 year showed a slight bias towards lower values. This bias stemmed from the inclusion of SARS-CoV-2 tests conducted between 40 and 90 days post-initial infection. Tests within this timeframe captured sporadic positive PCR results that did not signify genuine reinfections but rather reflected instances in which the initial infection persisted beyond 40 days or in which PCR positivity persisted because of residual non-viable viral fragments^54^. This source of bias, however, should not affect other estimates in this study as it is very rare for such false reinfections to occur after 90 days post-initial infection^21^.

A consequence of the test-negative design is that effectiveness estimates depend on the timing of the tests, specifically the interval between the previous infection and the SARS-CoV-2 test used in the study^15^. This can lead to seemingly contradictory results that are not necessarily indicative of true discrepancies. For instance, in the Omicron era analysis, the effectiveness against any reinfection (53.6%) was higher than that against symptomatic reinfection (45.4%). This might seem counterintuitive, as protection against symptomatic illness is typically expected to be higher^55^. However, this apparent discrepancy can be explained by the differential timing between the previous infection and the study test. The median time between the previous infection and the study test was 245 days for the analysis of any reinfection, whereas it was longer at 301 days for the analysis of symptomatic reinfection. This longer interval in the latter analysis resulted in greater waning of immune protection, leading to the observed difference in effectiveness estimates.

With the relatively young population of Qatar^17^, our findings may not be generalizable to other countries in which older citizens constitute a large proportion of the population. Whereas robust matching was implemented, the availability of data prevented matching on other factors such as geography or occupation. However, being essentially a city state, infection incidence in Qatar was broadly distributed across neighbourhoods. Nationality, age and sex provide a powerful proxy for socioeconomic status in this country^17^, and thus matching by these factors may have also, at least partially, controlled for other factors such as occupation. This matching approach has been previously investigated in studies of different epidemiologic designs, and using control groups to test for null effects^56–59^. These studies have supported that this matching prescription effectively controls for differences in infection exposure^56–59^.

However, bias in real-world data can arise unexpectedly or from unknown sources, such as subtle differences in test-seeking behaviour, changes in testing patterns because of policy shifts, variations in test accessibility or differences in the tendency to get tested between individuals who have recovered from a previous infection and those who have not been infected or whose previous infection was undocumented. Nevertheless, the further analyses investigating the impact of different sources of bias and the replication of the entire study using a different study design—a cohort study—confirmed and validated the findings.

This study has strengths. First, it was conducted on a national scale, encompassing a diverse population based on national backgrounds, and used extensive and validated databases established through numerous SARS-CoV-2 infection studies. Second, controls were selected from the entire national population, and exact matching was used to ensure rigorous pairing of cases and controls. Third, the study design controlled for vaccination status, another strength of the test-negative design, allowing for differentiation between the effects of previous infection and vaccination. Effectiveness estimates were also obtained for both unvaccinated and vaccinated populations, and the results of these analyses were consistent with each other and with the main analysis for the entire population. Fourth, using distinct reinfection window definitions of 90 versus 40 days resulted in considerably different analysis datasets. Yet, the effectiveness estimates remained consistent across these varying criteria. Finally, the findings were corroborated through further analyses and by a complete replication using a distinct epidemiological study design.

In conclusion, a stark contrast is found in the protective effect of natural infection against SARS-CoV-2 reinfection between the pre-Omicron and Omicron eras. Before Omicron emergence, natural infection provided robust and enduring protection against reinfection. However, during the Omicron era, this protection was strong only among recently infected individuals, rapidly declining and diminishing within 1 year. These contrasting patterns may suggest differing evolutionary pressures acting on the virus. In the pre-Omicron era, intrinsic transmissibility drove viral adaptation, whereas the widespread immunity acquired by the end of the first Omicron wave shifted the evolutionary pressure towards immune escape. This highlights the dynamic interplay between viral evolution and host immunity, necessitating continued monitoring of the virus and its evolution, as well as periodic updates of SARS-CoV-2 vaccines to restore immunity and counter continuing viral immune evasion.

## Methods

### Oversight

The institutional review boards at Hamad Medical Corporation and Weill Cornell Medicine–Qatar approved this retrospective study with a waiver of informed consent. The study was reported according to the Strengthening the Reporting of Observational Studies in Epidemiology (STROBE) guidelines (Extended Data Table 3).

### Study population and data sources

This study was conducted on the population of Qatar before and after the introduction of the Omicron variant on 19 December 2021 (ref. ^16^). The first analysis assessed the effectiveness of pre-Omicron infection in preventing reinfection with a pre-Omicron virus between 5 February 2020 (the onset of the COVID-19 pandemic in Qatar^17^) and 18 December 2021. The second analysis assessed the effectiveness of Omicron infection in preventing reinfection with an Omicron virus between 19 December 2021 and 12 February 2024 (marking the end of the study).

The data encompassed the national, federated databases for COVID-19 laboratory testing, vaccination, hospitalization and death, retrieved from the integrated, nationwide, digital-health information platform (Supplementary Methods section 2). These databases have captured SARS-CoV-2-related data with no missing information since the onset of the pandemic, including all PCR tests regardless of location or facility, and, from 5 January 2022, all medically supervised rapid antigen tests (Supplementary Methods section 3). SARS-CoV-2 testing was extensive in Qatar until 31 October 2022, with nearly 5% of the population being tested every week, primarily for routine purposes such as screening or meeting travel-related requirements^56,60^. Subsequently, testing rates decreased, with less than 1% of the population being tested per week^61^. Most infections during the pandemic were diagnosed through routine testing rather than symptomatic presentation^56,60^.

Qatar launched its COVID-19 vaccination programme in December 2020, using messenger RNA vaccines and prioritizing individuals on the basis of coexisting conditions and age criteria^56,59^. COVID-19 vaccination was provided free of charge, regardless of citizenship or residency status, and was nationally tracked^56,59^. Demographic information, including sex, age and nationality, was extracted from the records of the national health registry. Qatar shows demographic diversity, with 89% of its residents being expatriates from more than 150 countries^17^. Detailed descriptions of Qatar’s population and national databases have been previously reported^17,32,51,53,56,60,62^.

### Study design

The effectiveness of natural infection against reinfection was estimated using the test-negative, case-control study design, which compares the odds of previous infection among SARS-CoV-2-positive tests (cases) to that among SARS-CoV-2-negative tests (controls)^11,12,15,16,63,64^. The assessment was conducted both overall and by time since previous infection, using 3-month intervals.

Cases and controls were determined on the basis of the results of SARS-CoV-2 tests conducted during each analysis period. Cases were defined as SARS-CoV-2-positive tests, whereas controls were defined as SARS-CoV-2-negative tests. SARS-CoV-2 reinfection is conventionally defined as a documented infection more than or equal to 90 days after a previous infection, to avoid misclassifying prolonged test positivity as reinfection with shorter time intervals^16,21,65,66^. Consequently, cases or controls preceded by SARS-CoV-2-positive tests within 90 days were excluded. Previous infection was defined as a SARS-CoV-2-positive test more than or equal to 90 days before the study test.

To comply with the non-differential healthcare-seeking behaviour assumption inherent to the test-negative study design^15,63,64^, only tests with a documented reason for testing were included in the analysis. In the Omicron era analysis, cases or controls preceded by a pre-Omicron infection were excluded from the analysis, as the research question pertained to the effectiveness of Omicron immunity, rather than pre-Omicron immunity, against Omicron reinfection. The protection provided by pre-Omicron immunity against Omicron reinfection has been previously investigated^14,16,60^.

In estimating effectiveness in preventing reinfection, cases and controls were matched exactly one-to-two by sex, 10-year age group, nationality, number of coexisting conditions (ranging from zero to more than or equal to six; Supplementary Methods section 1), number of vaccine doses (ranging from zero to more than or equal to four), calendar week of the SARS-CoV-2 test, method of testing (PCR or rapid antigen) and reason for testing. This matching strategy aimed to balance observed confounders that could potentially influence the risk of infection across the exposure groups^17,67–70^. The selection of matching factors was guided by findings from earlier studies on Qatar’s population^11,56–59,71^ and the need to comply with the non-differential healthcare-seeking behaviour assumption inherent to the test-negative design^15,63,64^. This requirement was met by matching by the calendar week of the SARS-CoV-2 test, method of testing and reason for testing. In estimating effectiveness in preventing severe^72^, critical^72^ or fatal^73^ COVID-19 on reinfection, a one-to-five matching ratio was applied to enhance statistical precision.

Classification of severe^72^, critical^72^ and fatal^73^ COVID-19 followed the World Health Organization guidelines (Supplementary Methods section 4). The assessments were made by trained medical personnel independent of study investigators and using individual chart reviews. As part of the national protocol, each individual who had a SARS-CoV-2-positive test and concurrent COVID-19 hospital admission was subject to an infection severity assessment every 3 days until discharge or death, irrespective of hospital length of stay^26^. Individuals who progressed to severe, critical or fatal COVID-19 between the SARS-CoV-2-positive test and the end of this study were classified on the basis of their worst outcome, starting with death, followed by critical disease and then severe disease.

The variant status of infections was determined on the basis of the dominant variant at the time of infection diagnosis. Infections were categorized as pre-Omicron or Omicron. The duration of dominance for every variant throughout the pandemic was determined using Qatar’s variant genomic surveillance^74–76^ (Extended Data Fig. 3), which includes viral genome sequencing^74^ and multiplex real-time quantitative PCR with reverse transcription (RT–qPCR) variant screening^75^ of weekly collected random positive clinical samples (Supplementary Methods section 3). Once the first massive Omicron wave started, virtually all infections were due to Omicron, as it displaced all pre-Omicron variants^16,60,62^.

### Statistical analysis

All records of SARS-CoV-2 testing were examined for the selection of cases and controls, but only matched samples were analysed. Cases and controls were described using frequency distributions and measures of central tendency and compared using standardized mean differences. A standardized mean difference of less than or equal to 0.1 indicated adequate matching^77^.

Odds ratios (ORs), comparing odds of previous infection among cases versus controls, and associated 95% CIs were derived using conditional logistic regression. Analyses stratified by time since previous infection considered the date for the most recent documented infection. CIs were not adjusted for multiplicity and interactions were not investigated. The reference group for all estimates comprised individuals with no documented previous infection.

Effectiveness measures and associated 95% CIs were calculated as 1-OR of previous infection among cases versus controls if the OR was less than one, and as 1/OR-1 if the OR was more than or equal to one (refs. ^15,32,63,78^). This approach ensured a symmetric scale for both negative and positive effectiveness, spanning from −100 to 100%, resulting in a clear and meaningful interpretation of effectiveness, regardless of the value being positive or negative.

In addition to estimating effectiveness of previous infection in preventing reinfection, regardless of symptoms, effectiveness was also assessed specifically against symptomatic reinfection. This was accomplished by restricting the analysis to tests performed owing to clinical suspicion, indicating the presence of symptoms consistent with a respiratory tract infection.

Subgroup analyses were performed, considering only unvaccinated and vaccinated individuals, respectively. A sensitivity analysis was undertaken by redefining SARS-CoV-2 reinfection as a documented infection occurring more than or equal to 40 days after a previous infection, instead of the conventional more than or equal to 90 days. This adjustment was informed by a recent analysis suggesting the adequacy of a 40-day time window to define reinfection^21^. Statistical analyses were conducted in STATA/SE version v.18.0 (Stata).

### Further validation analyses

#### Previous infection misclassification

Under-ascertainment of infection introduces misclassification of previous infection status into the test-negative design used in this study, potentially biasing the estimates^15^. To assess the impact of under-ascertainment on estimates of waning immune protection, mathematical modelling simulations were conducted by extending our previous work on the test-negative design methodology^15^. These simulations evaluated the impact of an infection ascertainment rate of only 10%, indicating that 90% of SARS-CoV-2 infections are undocumented and would thus be misclassified. The model incorporated a gradual (linear) waning of the protective effect of infection against reinfection. Analyses were performed for both pre-Omicron and Omicron waning patterns, with immune protection durations set at 3 years for the pre-Omicron era^14^ and 1 year for the Omicron era. The results were reported at the 2-year mark from the onset of both the pre-Omicron and Omicron pandemic phases. A detailed description of the model, its methods and previous analyses can be found elsewhere^15^.

#### Coexisting conditions misclassification

Coexisting conditions were identified by analysing electronic health record encounters for each individual within the national public healthcare system’s database (Supplementary Methods section 1). However, this approach may not capture all conditions, as some may be undiagnosed or diagnosed at private facilities with unavailable records. To assess the impact of this potential bias on the estimated effectiveness of infection against reinfection, a sensitivity analysis was conducted in which matching by the number of coexisting conditions was entirely removed, simulating a scenario of complete under-ascertainment of these conditions.

#### Validation using a cohort study design

This study used the test-negative design^15^. To validate the findings, two extra national, matched, retrospective cohort studies—one for the pre-Omicron era and another for the Omicron era—were conducted. Each study compared the incidence of infection and severe forms of COVID-19 in two national cohorts: individuals with documented primary SARS-CoV-2 infection (primary-infection cohort) and uninfected individuals (uninfected cohort).

The first study estimated the effectiveness of a pre-Omicron primary infection in preventing reinfection with a pre-Omicron virus, and the second study estimated the effectiveness of an Omicron primary infection in preventing reinfection with an Omicron virus. Both studies were conducted over the same study durations as in the main analysis using the test-negative design. Incidence of infection was defined as any PCR-positive or rapid antigen-positive test after the start of follow-up, irrespective of symptomatic presentation.

Cohorts were matched exactly one-to-one by sex, 10-year age group, nationality, number of coexisting conditions, number of vaccine doses at the start of the follow-up, calendar week of the SARS-CoV-2-positive test for the primary-infection cohort and SARS-CoV-2-negative test for the uninfected cohort, method of testing (PCR or rapid antigen) and reason for testing. In both studies, individuals in the matched primary-infection cohort may have contributed follow-up time in the uninfected cohort before their primary infection and subsequently contributed follow-up time as part of the primary-infection cohort after contracting the infection.

Follow-up for each matched pair started 90 days after the primary infection for the individual in the primary-infection cohort. To ensure exchangeability, both members of each matched pair were censored at the earliest occurrence of receiving an extra vaccine dose. Accordingly, individuals were followed until the first of any of the following events: a documented SARS-CoV-2 infection, a new vaccine dose for the individual in either the primary-infection cohort or the uninfected cohort (with matched-pair censoring), death or the administrative end of follow-up, which was set at the end of the study or 15 months after the primary infection, whichever came first.

The overall adjusted hazard ratio (aHR), comparing the incidence of SARS-CoV-2 infection (or severe forms of COVID-19) between the cohorts, and the corresponding 95% CI, were calculated using Cox regression models with adjustment for the matching factors and testing rate in the cohorts, using the Stata v.18.0 stcox command. This adjustment was implemented to ensure precise and unbiased estimation of the standard variance^79^.

The overall aHR provides a weighted average of the time-varying hazard ratio^80^. To explore differences in the risk of infection (or severe forms of COVID-19) over time, the aHR was also estimated by 3-month intervals from the start of follow-up using separate Cox regressions, with ‘failure’ restricted to specific time intervals.

Effectiveness of infection against reinfection and against severe, critical or fatal COVID-19, along with the associated 95% CIs, were derived from the aHR as 1-aHR if the aHR was less than one and as 1/aHR-1 if the aHR was more than or equal to one (refs. ^32,78^). This approach ensured a symmetric scale for both negative and positive effectiveness, spanning from −100 to 100%, resulting in a meaningful interpretation of effectiveness, regardless of the value being positive or negative.

Statistical analyses were performed using Stata/SE v.18.0 (Stata). Further details on this type of cohort study design can be found in our previous publications, which used also the same national databases to estimate the effectiveness of infection against reinfection or the effectiveness of vaccination against infection^4,8,10,14,15,30,32,33,45,59,61,62,71,81–83^.

### Reporting summary

Further information on research design is available in the Nature Portfolio Reporting Summary linked to this article.

## Online content

Any methods, additional references, Nature Portfolio reporting summaries, source data, extended data, supplementary information, acknowledgements, peer review information; details of author contributions and competing interests; and statements of data and code availability are available at 10.1038/s41586-024-08511-9.

## Supplementary information

Supplementary Methods Supplementary Methods include sections 1–4 covering respectively, the classification of coexisting conditions, study population and data sources, laboratory methods and variant ascertainment, and COVID-19 severity, criticality and fatality classification.

Reporting Summary

Peer Review file

## Figures and Tables

**Extended Data Table 1:**
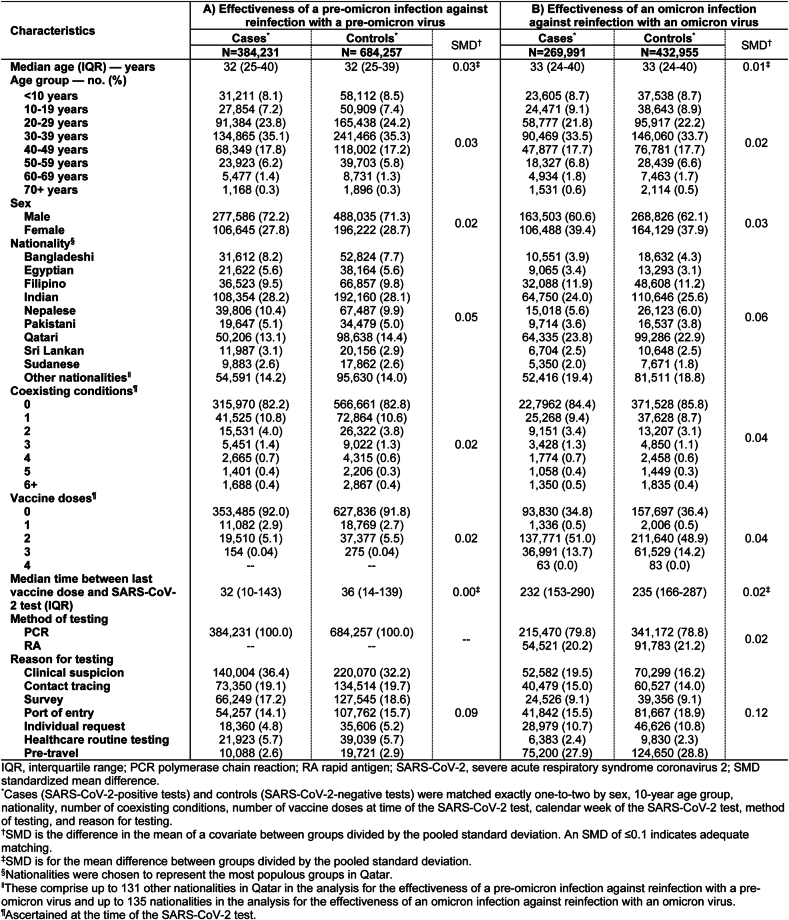
Study population characteristics

Characteristics of matched cases and controls in samples used to estimate the effectiveness of **a**) a pre-omicron infection in preventing reinfection with a pre-omicron virus and **b**) an omicron infection in preventing reinfection with an omicron virus.

**Extended Data Table 2:**
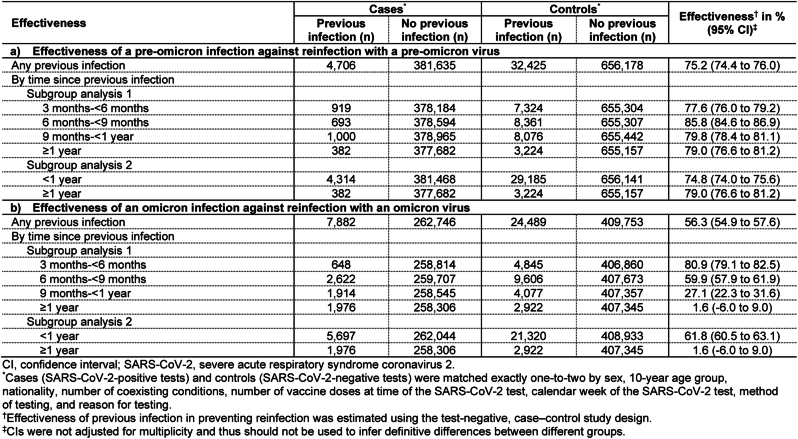
Sensitivity analysis employing 40-day time window to define reinfection

Effectiveness of a **a**) pre-omicron infection in preventing reinfection with a pre-omicron virus and **b**) an omicron infection in preventing reinfection with an omicron virus, assuming a 40-day time window for defining reinfection instead of the conventional 90-day time window.

**Extended Data Table 3:**
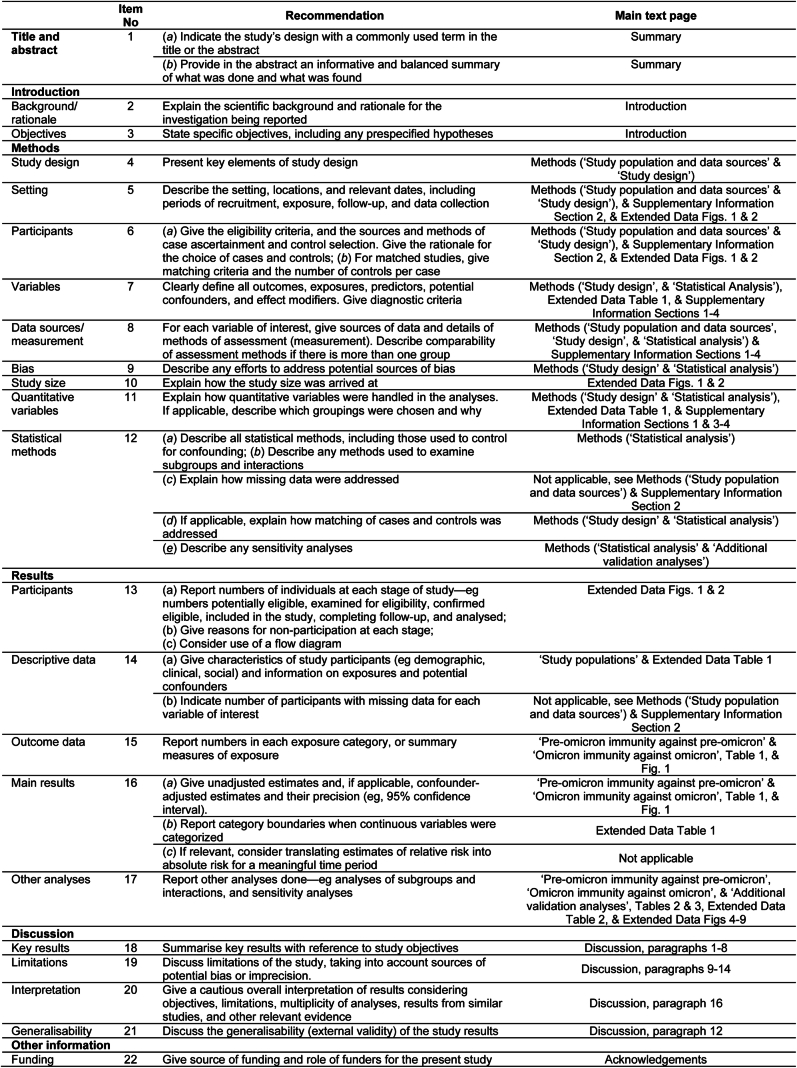
Reporting checklist

Strengthening the Reporting of Observational Studies in Epidemiology (STROBE) checklist for case-control studies.

## Data Availability

The National Coronavirus Disease 2019 (COVID-19) dataset used in this study is a property of the Qatar Ministry of Public Health that was provided to the researchers through a restricted-access agreement that prevents sharing the dataset with a third party or publicly. This dataset encompasses the National COVID-19 Testing Database, the National COVID-19 Vaccination Database, the National COVID-19 Severity Database, and the National Mortality Database. These data are available under restricted access for preservation of confidentiality of patient data. Access can be obtained through a direct application for data access to Her Excellency the Minister of Public Health (https://www.moph.gov.qa/english/OurServices/eservices/Pages/Governmental-HealthCommunication-Center.aspx). Data were available to authors through .csv files in which information has been downloaded from the CERNER database system (no links/accession codes were available to authors). The raw data are protected and are not available owing to data privacy laws. Aggregate data are available within the paper and its Supplementary information.

## Code availability

Standard epidemiological analyses were conducted using standard commands in STATA/SE v.18.0. These commands have been published at: https://github.com/IDEGWCMQ/TestNegCode/blob/main/TestNegCode.txt.

## References

[1] Markov, P. V. et al. The evolution of SARS-CoV-2. *Nat. Rev. Microbiol.* **21**, 361–379 (2023). 37020110 10.1038/s41579-023-00878-2

[2] Roemer, C. et al. SARS-CoV-2 evolution in the omicron era. *Nat. Microbiol.* **8**, 1952–1959 (2023). 37845314 10.1038/s41564-023-01504-w

[3] Subissi, L. et al. An early warning system for emerging SARS-CoV-2 variants. *Nat. Med.* **28**, 1110–1115 (2022). 35637337 10.1038/s41591-022-01836-w PMC11346314

[4] Abu-Raddad, L. J. et al. Introduction and expansion of the SARS-CoV-2 B.1.1.7 variant and reinfections in Qatar: a nationally representative cohort study. *PLoS Med.* **18**, e1003879 (2021). 34914711 10.1371/journal.pmed.1003879 PMC8726501

[5] Abu-Raddad, L. J. et al. Severity, criticality, and fatality of the SARS-CoV-2 Beta variant. *Clin. Infect. Dis*. 10.1093/cid/ciab909 (2021). 10.1093/cid/ciab909 PMC9402694 34657152

[6] Butt, A. A. et al. Severity of illness in persons infected with the SARS-CoV-2 Delta variant vs Beta variant in Qatar. *JAMA Intern. Med.* **182**, 197–205, (2022). 34935861 10.1001/jamainternmed.2021.7949 PMC8696690

[7] Covid Forecasting Team. Past SARS-CoV-2 infection protection against re-infection: a systematic review and meta-analysis. *Lancet* **401**, 833–842 (2023). 36930674 10.1016/S0140-6736(22)02465-5 PMC9998097

[8] Abu-Raddad, L. J. et al. SARS-CoV-2 antibody-positivity protects against reinfection for at least seven months with 95% efficacy. *EClinMed.* **35**, 100861 (2021). 10.1016/j.eclinm.2021.100861 PMC8079668 33937733

[9] Bobrovitz, N. et al. Protective effectiveness of previous SARS-CoV-2 infection and hybrid immunity against the omicron variant and severe disease: a systematic review and meta-regression. *Lancet Infect. Dis.* **23**, 556–567 (2023). 36681084 10.1016/S1473-3099(22)00801-5 PMC10014083

[10] Chemaitelly, H., Bertollini, R., Abu-Raddad, L. J. & National Study Group for COVID Epidemiology. Efficacy of natural immunity against SARS-CoV-2 reinfection with the beta variant. *N. Engl. J. Med.* **385**, 2585–2586 (2021). 34910864 10.1056/NEJMc2110300 PMC8693689

[11] Chemaitelly, H. et al. Protection against reinfection with the omicron BA.2.75 subvariant. *N. Engl. J. Med.* **388**, 665–667 (2023). 36652342 10.1056/NEJMc2214114 PMC9878583

[12] Altarawneh, H. N. et al. Protective effect of previous SARS-CoV-2 infection against omicron BA.4 and BA.5 subvariants. *N. Engl. J. Med.* **387**, 1620–1622 (2022). 36198139 10.1056/NEJMc2209306 PMC9559315

[13] Chemaitelly, H. et al. Protection of natural infection against reinfection with SARS-CoV-2 JN.1 variant. *J. Travel Med.*10.1093/jtm/taae053 (2024). 10.1093/jtm/taae053 PMC11149714 38591115

[14] Chemaitelly, H. et al. Duration of immune protection of SARS-CoV-2 natural infection against reinfection. *J. Travel Med.*10.1093/jtm/taac109 (2022). 10.1093/jtm/taac109 PMC9619565 36179099

[15] Ayoub, H. H. et al. Estimating protection afforded by prior infection in preventing reinfection: applying the test-negative study design. *Am. J. Epidemiol*. 10.1093/aje/kwad239 (2023). 10.1093/aje/kwad239 PMC11145912 38061757

[16] Altarawneh, H. N. et al. Protection against the omicron variant from previous SARS-CoV-2 infection. *N. Engl. J. Med.* **386**, 1288–1290 (2022). 35139269 10.1056/NEJMc2200133 PMC8849180

[17] Abu-Raddad, L. J. et al. Characterizing the Qatar advanced-phase SARS-CoV-2 epidemic. *Sci. Rep.* **11**, 6233 (2021). 33737535 10.1038/s41598-021-85428-7 PMC7973743

[18] Bouhaddou, M. et al. SARS-CoV-2 variants evolve convergent strategies to remodel the host response. *Cell* **186**, 4597–4614 e4526 (2023). 37738970 10.1016/j.cell.2023.08.026 PMC10604369

[19] Carabelli, A. M. et al. SARS-CoV-2 variant biology: immune escape, transmission and fitness. *Nat. Rev. Microbiol.* **21**, 162–177 (2023). 36653446 10.1038/s41579-022-00841-7 PMC9847462

[20] Willett, B. J. et al. SARS-CoV-2 omicron is an immune escape variant with an altered cell entry pathway. *Nat. Microbiol.* **7**, 1161–1179 (2022). 35798890 10.1038/s41564-022-01143-7 PMC9352574

[21] Chemaitelly, H. et al. Addressing bias in the definition of SARS-CoV-2 reinfection: implications for underestimation. *Front. Med.* **11**, 1363045 (2024). 10.3389/fmed.2024.1363045 PMC10961414 38529118

[22] du Plessis, L. et al. Establishment and lineage dynamics of the SARS-CoV-2 epidemic in the UK. *Science* **371**, 708–712 (2021). 33419936 10.1126/science.abf2946 PMC7877493

[23] Davies, N. G. et al. Estimated transmissibility and impact of SARS-CoV-2 lineage B.1.1.7 in England. *Science*10.1126/science.abg3055 (2021). 10.1126/science.abg3055 PMC8128288 33658326

[24] Luo, C. H. et al. Infection with the SARS-CoV-2 delta variant is associated with higher recovery of infectious virus compared to the alpha variant in both unvaccinated and vaccinated individuals. *Clin. Infect. Dis.*10.1093/cid/ciab986 (2021). 10.1093/cid/ciab986 PMC8903351 34922338

[25] Qassim, S. H. et al. Effects of SARS-CoV-2 Alpha, Beta, and Delta variants, age, vaccination, and prior infection on infectiousness of SARS-CoV-2 infections. *Front. Immunol.* **13**, 984784 (2022). 36177014 10.3389/fimmu.2022.984784 PMC9513583

[26] Chemaitelly, H. et al. Turning point in COVID-19 severity and fatality during the pandemic: a national cohort study in Qatar. *BMJ Public Health* **1**, e000479 (2023). 40017867 10.1136/bmjph-2023-000479 PMC11812731

[27] Cao, Y. et al. Imprinted SARS-CoV-2 humoral immunity induces convergent Omicron RBD evolution. *Nature* **614**, 521–529 (2023). 36535326 10.1038/s41586-022-05644-7 PMC9931576

[28] Ito, J. et al. Convergent evolution of SARS-CoV-2 omicron subvariants leading to the emergence of BQ.1.1 variant. *Nat. Commun.* **14**, 2671 (2023). 37169744 10.1038/s41467-023-38188-z PMC10175283

[29] Reynolds, C. J. et al. Immune boosting by B.1.1.529 (omicron) depends on previous SARS-CoV-2 exposure. *Science* **377**, eabq1841 (2022). 35699621 10.1126/science.abq1841 PMC9210451

[30] Chemaitelly, H. et al. History of primary-series and booster vaccination and protection against omicron reinfection. *Sci. Adv.* **9**, eadh0761 (2023). 37792951 10.1126/sciadv.adh0761 PMC10550237

[31] Roltgen, K. et al. Immune imprinting, breadth of variant recognition, and germinal center response in human SARS-CoV-2 infection and vaccination. *Cell* **185**, 1025–1040 e1014 (2022). 35148837 10.1016/j.cell.2022.01.018 PMC8786601

[32] Chemaitelly, H. et al. Long-term COVID-19 booster effectiveness by infection history and clinical vulnerability and immune imprinting: a retrospective population-based cohort study. *Lancet Infect. Dis.* **23**, 816–827 (2023). 36913963 10.1016/S1473-3099(23)00058-0 PMC10079373

[33] Chemaitelly, H. et al. Immune imprinting and protection against repeat reinfection with SARS-CoV-2. *N. Engl. J. Med.*10.1056/NEJMc2211055 (2022). 10.1056/NEJMc2211055 PMC9634858 36223534

[34] Doria-Rose, N. et al. Antibody persistence through 6 months after the second dose of mRNA-1273 vaccine for COVID-19. *N. Engl. J. Med.* **384**, 2259–2261 (2021). 33822494 10.1056/NEJMc2103916 PMC8524784

[35] Gilbert, P. B. et al. A COVID-19 milestone attained - a correlate of protection for vaccines. *N. Engl. J. Med.* **387**, 2203–2206 (2022). 36507702 10.1056/NEJMp2211314

[36] Sette, A. & Crotty, S. Adaptive immunity to SARS-CoV-2 and COVID-19. *Cell* **184**, 861–880 (2021). 33497610 10.1016/j.cell.2021.01.007 PMC7803150

[37] Wherry, E. J. & Barouch, D. H. T cell immunity to COVID-19 vaccines. *Science* **377**, 821–822 (2022). 35981045 10.1126/science.add2897

[38] Altmann, D. M. & Boyton, R. J. Arming up against omicron subvariants. *Cell Host Microbe.* **32**, 147–148 (2024). 38359794 10.1016/j.chom.2024.01.010

[39] Abu-Raddad, L. J., Chemaitelly, H., Bertollini, R. & National Study Group for COVID Epidemiology. Severity of SARS-CoV-2 reinfections as compared with primary infections. *N. Engl. J. Med.* **385**, 2487–2489 (2021). 34818474 10.1056/NEJMc2108120 PMC8631440

[40] Abu-Raddad, L. J. et al. Assessment of the risk of Severe Acute Respiratory Syndrome Coronavirus 2 (SARS-CoV-2) reinfection in an intense reexposure setting. *Clin. Infect. Dis.* **73**, e1830–e1840 (2021). 33315061 10.1093/cid/ciaa1846 PMC7799253

[41] Altarawneh, H. N. et al. Effects of previous infection, vaccination, and hybrid immunity against symptomatic Alpha, Beta, and Delta SARS-CoV-2 infections: an observational study. *EBioMed.* **95**, 104734 (2023). 10.1016/j.ebiom.2023.104734 PMC10404859 37515986

[42] Chemaitelly, H. et al. Duration of mRNA vaccine protection against SARS-CoV-2 Omicron BA.1 and BA.2 subvariants in Qatar. *Nat. Commun.* **13**, 3082 (2022). 35654888 10.1038/s41467-022-30895-3 PMC9163167

[43] Higdon, M. M. et al. Duration of effectiveness of vaccination against COVID-19 caused by the omicron variant. *Lancet Infect. Dis.* **22**, 1114–1116 (2022). 35752196 10.1016/S1473-3099(22)00409-1 PMC9221361

[44] Andrews, N. et al. COVID-19 vaccine effectiveness against the Omicron (B.1.1.529) variant. *N. Engl. J. Med.* **386**, 1532–1546 (2022). 35249272 10.1056/NEJMoa2119451 PMC8908811

[45] Chemaitelly, H. et al. COVID-19 vaccine protection among children and adolescents in Qatar. *N. Engl. J. Med.* **387**, 1865–1876 (2022). 36322837 10.1056/NEJMoa2210058 PMC9644642

[46] Qassim, S. H. et al. Population immunity of natural infection, primary-series vaccination, and booster vaccination in Qatar during the COVID-19 pandemic: an observational study. *EClinMed.* **62**, 102102 (2023). 10.1016/j.eclinm.2023.102102 PMC10393554 37533414

[47] Kissler, S. M., Tedijanto, C., Goldstein, E., Grad, Y. H. & Lipsitch, M. Projecting the transmission dynamics of SARS-CoV-2 through the postpandemic period. *Science* **368**, 860–868 (2020). 32291278 10.1126/science.abb5793 PMC7164482

[48] Lavine, J. S., Bjornstad, O. N. & Antia, R. Immunological characteristics govern the transition of COVID-19 to endemicity. *Science* **371**, 741–745 (2021). 33436525 10.1126/science.abe6522 PMC7932103

[49] Ferguson, N. M., Galvani, A. P. & Bush, R. M. Ecological and immunological determinants of influenza evolution. *Nature* **422**, 428–433 (2003). 12660783 10.1038/nature01509

[50] Patel, M. M., York, I. A., Monto, A. S., Thompson, M. G. & Fry, A. M. Immune-mediated attenuation of influenza illness after infection: opportunities and challenges. *Lancet Microbe.* **2**, e715–e725 (2021). 35544110 10.1016/S2666-5247(21)00180-4

[51] Chemaitelly, H. et al. Short- and longer-term all-cause mortality among SARS-CoV-2- infected individuals and the pull-forward phenomenon in Qatar: a national cohort study. *Int. J. Infect. Dis.* **136**, 81–90 (2023). 37717648 10.1016/j.ijid.2023.09.005

[52] Seedat, S. et al. SARS-CoV-2 infection hospitalization, severity, criticality, and fatality rates in Qatar. *Sci. Rep.* **11**, 18182 (2021). 34521903 10.1038/s41598-021-97606-8 PMC8440606

[53] AlNuaimi, A. A. et al. All-cause and COVID-19 mortality in Qatar during the COVID-19 pandemic. *BMJ Glob. Health* **8**, e012291 (2023). 37142299 10.1136/bmjgh-2023-012291 PMC10163334

[54] Abu-Raddad, L. J. et al. Two prolonged viremic SARS-CoV-2 infections with conserved viral genome for two months. *Infect. Genet. Evol.* **88**, 104684 (2021). 33352320 10.1016/j.meegid.2020.104684 PMC7759339

[55] Sukik, L. et al. Effectiveness of two and three doses of COVID-19 mRNA vaccines against infection, symptoms, and severity in the pre-omicron era: a time-dependent gradient. *Vaccine*10.1016/j.vaccine.2024.04.026 (2024). 10.1016/j.vaccine.2024.04.026 38616439

[56] Chemaitelly, H. et al. Waning of BNT162b2 vaccine protection against SARS-CoV-2 infection in Qatar. *N. Engl. J. Med.* **385**, e83 (2021). 34614327 10.1056/NEJMoa2114114 PMC8522799

[57] Chemaitelly, H. et al. mRNA-1273 COVID-19 vaccine effectiveness against the B.1.1.7 and B.1.351 variants and severe COVID-19 disease in Qatar. *Nat. Med.* **27**, 1614–1621 (2021). 34244681 10.1038/s41591-021-01446-y

[58] Abu-Raddad, L. J., Chemaitelly, H., Bertollini, R. & National Study Group for COVID Vaccination. Waning mRNA-1273 vaccine effectiveness against SARS-CoV-2 infection in Qatar. *N. Engl. J. Med.* **386**, 1091–1093 (2022). 35081294 10.1056/NEJMc2119432 PMC8809505

[59] Abu-Raddad, L. J., Chemaitelly, H., Bertollini, R. & National Study Group for COVID Vaccination. Effectiveness of mRNA-1273 and BNT162b2 vaccines in Qatar. *N. Engl. J. Med.* **386**, 799–800 (2022). 35045222 10.1056/NEJMc2117933 PMC8796790

[60] Altarawneh, H. N. et al. Effects of previous infection and vaccination on symptomatic omicron infections. *N. Engl. J. Med.* **387**, 21–34 (2022). 35704396 10.1056/NEJMoa2203965 PMC9258753

[61] Chemaitelly, H. et al. Bivalent mRNA-1273.214 vaccine effectiveness against SARS-CoV-2 omicron XBB* infections. *J. Travel Med.* **30**, taad106 (2023). 37555656 10.1093/jtm/taad106 PMC10481416

[62] Abu-Raddad, L. J. et al. Effect of mRNA vaccine boosters against SARS-CoV-2 omicron infection in Qatar. *N. Engl. J. Med.* **386**, 1804–1816 (2022). 35263534 10.1056/NEJMoa2200797 PMC8929389

[63] Jackson, M. L. & Nelson, J. C. The test-negative design for estimating influenza vaccine effectiveness. *Vaccine* **31**, 2165–2168 (2013). 23499601 10.1016/j.vaccine.2013.02.053

[64] Verani, J. R. et al. Case-control vaccine effectiveness studies: preparation, design, and enrollment of cases and controls. *Vaccine* **35**, 3295–3302 (2017). 28442231 10.1016/j.vaccine.2017.04.037 PMC7007298

[65] Pilz, S., Theiler-Schwetz, V., Trummer, C., Krause, R. & Ioannidis, J. P. A. SARS-CoV-2 reinfections: overview of efficacy and duration of natural and hybrid immunity. *Environ. Res.* **209**, 112911 (2022). 35149106 10.1016/j.envres.2022.112911 PMC8824301

[66] Kojima, N., Shrestha, N. K. & Klausner, J. D. A systematic review of the protective effect of prior SARS-CoV-2 infection on repeat infection. *Eval. Health Prof.* **44**, 327–332 (2021). 34592838 10.1177/01632787211047932 PMC8564250

[67] Ayoub, H. H. et al. Mathematical modeling of the SARS-CoV-2 epidemic in Qatar and its impact on the national response to COVID-19. *J. Glob. Health* **11**, 05005 (2021). 33643638 10.7189/jogh.11.05005 PMC7897910

[68] Coyle, P. V. et al. SARS-CoV-2 seroprevalence in the urban population of Qatar: an analysis of antibody testing on a sample of 112,941 individuals. *iScience* **24**, 102646 (2021). 34056566 10.1016/j.isci.2021.102646 PMC8142077

[69] Jeremijenko, A. et al. Herd immunity against Severe Acute Respiratory Syndrome Coronavirus 2 infection in 10 communities, Qatar. *Emerg. Infect. Dis.* **27**, 1343–1352 (2021). 33900174 10.3201/eid2705.204365 PMC8084480

[70] Al-Thani, M. H. et al. SARS-CoV-2 infection is at herd immunity in the majority segment of the population of Qatar. *Open Forum. Infect. Dis.* **8**, ofab221 (2021). 34458388 10.1093/ofid/ofab221 PMC8135898

[71] Chemaitelly, H. et al. Protection of omicron sub-lineage infection against reinfection with another omicron sub-lineage. *Nat. Commun.* **13**, 4675 (2022). 35945213 10.1038/s41467-022-32363-4 PMC9362989

[72] World Health Organization.* Living Guidance for Clinical Management of COVID-19* https://www.who.int/publications/i/item/WHO-2019-nCoV-clinical-2021-2 (2023).

[73] World Health Organization. *International Guidelines for Certification and Classification (Coding) of COVID-19 as Cause of Death* https://www.who.int/publications/m/item/international-guidelines-for-certification-and-classification-(coding)-of-covid-19-as-cause-of-death (2023).

[74] Benslimane, F. M. et al. One year of SARS-CoV-2: genomic characterization of COVID-19 outbreak in Qatar. *Front. Cell Infect. Microbiol.* **11**, 768883 (2021). 34869069 10.3389/fcimb.2021.768883 PMC8637114

[75] Hasan, M. R. et al. Real-time SARS-CoV-2 genotyping by high-throughput multiplex PCR reveals the epidemiology of the variants of concern in Qatar. *Int. J. Infect. Dis.* **112**, 52–54 (2021). 34525398 10.1016/j.ijid.2021.09.006

[76] Saththasivam, J. et al. COVID-19 (SARS-CoV-2) outbreak monitoring using wastewater-based epidemiology in Qatar. *Sci. Total Environ.* **774**, 145608 (2021). 33607430 10.1016/j.scitotenv.2021.145608 PMC7870436

[77] Austin, P. C. Using the standardized difference to compare the prevalence of a binary variable between two groups in observational research. *Commun. Stat. Simul. Comput.* **38**, 1228–1234 (2009).

[78] Tseng, H. F. et al. Effectiveness of mRNA-1273 vaccination against SARS-CoV-2 omicron subvariants BA.1, BA.2, BA.2.12.1, BA.4, and BA.5. *Nat. Commun.* **14**, 189 (2023). 36635284 10.1038/s41467-023-35815-7 PMC9836332

[79] Sjolander, A. & Greenland, S. Ignoring the matching variables in cohort studies – when is it valid and why? *Stat. Med.* **32**, 4696–4708 (2013). 23761197 10.1002/sim.5879

[80] Stensrud, M. J. & Hernan, M. A. Why test for proportional hazards? *JAMA* **323**, 1401–1402 (2020). 32167523 10.1001/jama.2020.1267 PMC11983487

[81] Abu-Raddad, L. J. et al. Association of prior SARS-CoV-2 infection with risk of breakthrough infection following mRNA vaccination. *JAMA* **326**, 1930–1939 (2021). 34724027 10.1001/jama.2021.19623 PMC8561432

[82] Chemaitelly, H. et al. Protection from previous natural infection compared with mRNA vaccination against SARS-CoV-2 infection and severe COVID-19 in Qatar: a retrospective cohort study. *Lancet Microbe* **3**, e944–e955 (2022). 36375482 10.1016/S2666-5247(22)00287-7 PMC9651957

[83] Chemaitelly, H. et al. BNT162b2 antigen dose and SARS-CoV-2 omicron infection in adolescents. *Lancet Infect. Dis.* **23**, 276–277 (2023). 36738760 10.1016/S1473-3099(23)00005-1 PMC9891733

