# Supplementary material for: Differential protection against SARS-CoV-2 reinfection pre- and post-Omicron
Source: Nature. 2025 Feb 5;639(8056):1024–31. doi: 10.1038/s41586-024-08511-9 (PMC11946897; doi:10.1038/s41586-024-08511-9)
Supplement: Supplementary file 1 — Supplementary Methods include sections 1–4 covering respectively, the classification of coexisting conditions, study population and data sources, laboratory methods and variant ascertainment, and COVID-19 severity, criticality and fatality classification. [file 41586_2024_8511_MOESM1_ESM.pdf]

nature portfolio

https://doi.org/10.1038/s41586-024-08511-9

Supplementary information

Differential protection against SARS-CoV-2 reinfection pre- and post-Omicron

In the format provided by the
authors and unedited

Supplementary information

Supplementary Methods

Section 1. Classification of coexisting conditions

Coexisting conditions were ascertained and classified based on the ICD-10 codes for the conditions as recorded in the electronic health record encounters of each individual in the Cerner-system national database that includes all citizens and residents registered in the national and universal public healthcare system. The public healthcare system provides healthcare to the entire resident population of Qatar free of charge or at heavily subsidized costs, including prescription drugs. With the mass expansion of this sector in recent years, facilities have been built to cater to specific needs of subpopulations. For example, tens of facilities have been built, including clinics and hospitals, in localities with high density of craft and manual workers.[1]

All encounters for each individual were analysed to determine the coexisting-condition classification for that individual, as part of a recent national analysis to assess healthcare needs and resource allocation. The Cerner-system national database includes encounters starting from 2013, after this system was launched in Qatar. As long as each individual had at least one encounter with a specific coexisting-condition diagnosis since 2013, this person was classified with this coexisting condition.

Individuals who have coexisting conditions but never sought care in the public healthcare system, or seek care exclusively in private healthcare facilities, were classified as individuals with no coexisting condition due to absence of recorded encounters for them.

Section 2. Study population and data sources

Qatar's national and universal public healthcare system uses the Cerner-system advanced digital health platform to track all electronic health record encounters of each individual in the country, including all citizens and residents registered in the national and universal public healthcare system. Registration in the public healthcare system is mandatory for citizens and residents.

The databases analysed in this study are data-extract downloads from the Cerner-system that have been implemented on a regular schedule since the onset of pandemic by the Business Intelligence Unit at Hamad Medical Corporation (HMC). HMC is the national public healthcare provider in Qatar. At every download all severe acute respiratory syndrome coronavirus 2 (SARS-CoV-2) tests, coronavirus disease 2019 (COVID-19) vaccinations, hospitalizations related to COVID-19, and all death records regardless of cause are provided to the authors through .csv files. These databases have been analysed throughout the pandemic not only for study-related purposes, but also to provide policymakers with summary data and analytics to inform the national response.

Every health encounter in the Cerner-system is linked to a unique individual through the HMC Number that links all records for this individual at the national level. Databases were merged and analysed using the HMC Number to link all records whether for testing, vaccinations, hospitalizations, and deaths. All COVID-19-related healthcare was provided only in the public healthcare system. No private entity was permitted to provide COVID-19-related hospitalization. COVID-19 vaccination was also provided only through the public healthcare system. These health records were tracked throughout the COVID-19 pandemic using the Cerner system. This system has been implemented in 2013, before the onset of the pandemic. Therefore, we had the

health records related to this study for the full national cohort of citizens and residents throughout the pandemic.

Demographic details for every HMC Number (individual) such as sex, age, and nationality are collected upon issuing of the universal health card, based on the Qatar Identity Card, which is a mandatory requirement by the Ministry of Interior to every citizen and resident in the country. Data extraction from the Qatar Identity Card to the digital health platform is performed electronically through scanning techniques.

All SARS-CoV-2 testing in any facility in Qatar is tracked nationally in one database, the national testing database. This database covers all testing in all locations and facilities throughout the country, whether public or private. Every polymerase chain reaction (PCR) test and a proportion of the facility-based rapid antigen tests conducted in Qatar, regardless of location or setting, are classified on the basis of symptoms and the reason for testing (clinical symptoms, contact tracing, surveys or random testing campaigns, individual requests, routine healthcare testing, pre-travel, at port of entry, or other).

Before November 1, 2022, SARS-CoV-2 testing in Qatar was done at a mass scale where about 5% of the population were tested every week.[2] Based on the distribution of the reason for testing up to October 31, 2022, most of the tests in Qatar were conducted for routine reasons, such as being travel-related, and about 75% of cases were diagnosed not because of appearance of symptoms, but because of routine testing.[2,3] Subsequently, testing rates decreased, with less than 1% of the population being tested per week.[4] All testing results in the national testing database during the present study were factored in the analyses of this study.

The first large omicron wave that peaked in January of 2022 was massive and strained the testing capacity in the country.[2,4-6] Accordingly, rapid antigen testing was introduced to relieve the

pressure on PCR testing. Implementation of this change in testing policy occurred quickly precluding incorporation of reason for testing in a large proportion of the rapid antigen tests. While the reason for testing is available for all PCR tests, it is not available for all rapid antigen tests. Availability of reason for testing for the rapid antigen tests also varied with time.

Rapid antigen test kits are available for purchase in pharmacies in Qatar, but outcome of home-based testing is not reported nor documented in the national databases. Since SARS-CoV-2-test outcomes were linked to specific public health measures, restrictions, and privileges, testing policy and guidelines stress facility-based testing as the core testing mechanism in the population. While facility-based testing is provided free of charge or at low subsidized costs, depending on the reason for testing, home-based rapid antigen testing is de-emphasized and not supported as part of national policy.

Qatar launched its COVID-19 vaccination program in December 2020, employing mRNA vaccines and prioritizing individuals based on coexisting conditions and age criteria.[3,7] COVID-19 vaccination was provided free of charge, regardless of citizenship or residency status, and was nationally tracked.[3,7]

Qatar has unusually young, diverse demographics, in that only 9% of its residents are ≥50 years of age, and 89% are expatriates from over 150 countries.[8,9] Further descriptions of the study population and these national databases were reported previously.[2,3,6,9-13]

Section 3. Laboratory methods and variant ascertainment.

Real-time reverse-transcription polymerase chain reaction testing

Nasopharyngeal and/or oropharyngeal swabs were collected for polymerase chain reaction (PCR) testing and placed in Universal Transport Medium (UTM). Aliquots of UTM were: 1) extracted on KingFisher Flex (Thermo Fisher Scientific, USA), MGISP-960 (MGI, China), or ExiPrep 96 Lite (Bioneer, South Korea) followed by testing with real-time reverse-transcription PCR (RT-qPCR) using TaqPath COVID-19 Combo Kits (Thermo Fisher Scientific, USA) on an ABI 7500 FAST (Thermo Fisher Scientific, USA); 2) tested directly on the Cepheid GeneXpert system using the Xpert Xpress SARS-CoV-2 (Cepheid, USA); or 3) loaded directly into a Roche cobas 6800 system and assayed with the cobas SARS-CoV-2 Test (Roche, Switzerland). The first assay targets the viral S, N, and ORF1ab gene regions. The second targets the viral N and E-gene regions, and the third targets the ORF1ab and E-gene regions.

All PCR testing was conducted at the Hamad Medical Corporation Central Laboratory or Sidra Medicine Laboratory, following standardized protocols.

Rapid antigen testing

Severe acute respiratory syndrome coronavirus 2 (SARS-CoV-2) antigen tests were performed on nasopharyngeal swabs using one of the following lateral flow antigen tests: Panbio COVID-19 Ag Rapid Test Device (Abbott, USA); SARS-CoV-2 Rapid Antigen Test (Roche, Switzerland); Standard Q COVID-19 Antigen Test (SD Biosensor, Korea); or CareStart COVID-19 Antigen Test (Access Bio, USA). All antigen tests were performed point-of-care according to each manufacturer's instructions at public or private hospitals and clinics throughout Qatar with prior authorization and training by the Ministry of Public Health (MOPH). Antigen test results

were electronically reported to the MOPH in real time using the Antigen Test Management System which is integrated with the national Coronavirus Disease 2019 (COVID-19) database.

Classification of infections by variant type

Surveillance for SARS-CoV-2 variants in Qatar is based on viral genome sequencing and multiplex RT-qPCR variant screening[14] of weekly collected random positive clinical samples,[3,15-19] complemented by deep sequencing of wastewater samples.[17,20,21] Further details on the viral genome sequencing and multiplex RT-qPCR variant screening throughout the SARS-CoV-2 waves in Qatar can be found in previous publications.[2,3,5,11,15-19,22-26]

Section 4. COVID-19 severity, criticality, and fatality classification.

Classification of Coronavirus Disease 2019 (COVID-19) case severity (acute-care hospitalizations),[27] criticality (intensive-care-unit hospitalizations),[27] and fatality[28] followed World Health Organization (WHO) guidelines. Assessments were made by trained medical personnel independent of study investigators and using individual chart reviews, as part of a national protocol applied to every hospitalized COVID-19 patient. Each hospitalized COVID-19 patient underwent an infection severity assessment every three days until discharge or death. We classified individuals who progressed to severe, critical, or fatal COVID-19 between the time of the documented infection and the end of the study based on their worst outcome, starting with death,[28] followed by critical disease,[27] and then severe disease.[27]

Severe COVID-19 disease was defined per WHO classification as a SARS-CoV-2 infected person with "oxygen saturation of <90% on room air, and/or respiratory rate of >30 breaths/minute in adults and children >5 years old (or ≥60 breaths/minute in children <2 months old or ≥50 breaths/minute in children 2-11 months old or ≥40 breaths/minute in children 1–5 years old), and/or signs of severe respiratory distress (accessory muscle use and inability to complete full sentences, and, in children, very severe chest wall indrawing, grunting, central cyanosis, or presence of any other general danger signs)".[27] Detailed WHO criteria for classifying Severe acute respiratory syndrome coronavirus 2 (SARS-CoV-2) infection severity can be found in the WHO technical report.[27]

Critical COVID-19 disease was defined per WHO classification as a SARS-CoV-2 infected person with "acute respiratory distress syndrome, sepsis, septic shock, or other conditions that would normally require the provision of life sustaining therapies such as mechanical ventilation

(invasive or non-invasive) or vasopressor therapy”.[27] Detailed WHO criteria for classifying SARS-CoV-2 infection criticality can be found in the WHO technical report.[27]

COVID-19 death was defined per WHO classification as “a death resulting from a clinically compatible illness, in a probable or confirmed COVID-19 case, unless there is a clear alternative cause of death that cannot be related to COVID-19 disease (e.g. trauma). There should be no period of complete recovery from COVID-19 between illness and death. A death due to COVID-19 may not be attributed to another disease (e.g. cancer) and should be counted independently of preexisting conditions that are suspected of triggering a severe course of COVID-19”. Detailed WHO criteria for classifying COVID-19 death can be found in the WHO technical report.[28]

References

1 Al-Thani, M. H. *et al.* SARS-CoV-2 Infection Is at Herd Immunity in the Majority Segment of the Population of Qatar. *Open Forum Infect Dis* **8**, ofab221, doi:10.1093/ofid/ofab221 (2021).

2 Altarawneh, H. N. *et al.* Effects of Previous Infection and Vaccination on Symptomatic Omicron Infections. *N Engl J Med* **387**, 21-34, doi:10.1056/NEJMoa2203965 (2022).

3 Chemaitelly, H. *et al.* Waning of BNT162b2 Vaccine Protection against SARS-CoV-2 Infection in Qatar. *N Engl J Med* **385**, e83, doi:10.1056/NEJMoa2114114 (2021).

4 Chemaitelly, H. *et al.* Bivalent mRNA-1273.214 vaccine effectiveness against SARS-CoV-2 omicron XBB* infections. *J Travel Med* **30**, doi:10.1093/jtm/taad106 (2023).

5 Altarawneh, H. N. *et al.* Protection against the Omicron Variant from Previous SARS-CoV-2 Infection. *N Engl J Med* **386**, 1288-1290, doi:10.1056/NEJMc2200133 (2022).

6 Chemaitelly, H. *et al.* Long-term COVID-19 booster effectiveness by infection history and clinical vulnerability and immune imprinting: a retrospective population-based cohort study. *Lancet Infect Dis* **23**, 816-827, doi:10.1016/S1473-3099(23)00058-0 (2023).

7 Abu-Raddad, L. J., Chemaitelly, H., Bertollini, R. & National Study Group for Covid Vaccination. Effectiveness of mRNA-1273 and BNT162b2 Vaccines in Qatar. *N Engl J Med* **386**, 799-800, doi:10.1056/NEJMc2117933 (2022).

8 Planning and Statistics Authority-State of Qatar. Qatar Monthly Statistics. Available from: https://www.psa.gov.qa/en/pages/default.aspx. Accessed on: May 26, 2020. (2020).

9 Abu-Raddad, L. J. *et al.* Characterizing the Qatar advanced-phase SARS-CoV-2 epidemic. *Sci Rep* **11**, 6233, doi:10.1038/s41598-021-85428-7 (2021).

10 Chemaitelly, H., Bertollini, R., Abu-Raddad, L. J. & National Study Group for Covid Epidemiology. Efficacy of Natural Immunity against SARS-CoV-2 Reinfection with the Beta Variant. *N Engl J Med* **385**, 2585-2586, doi:10.1056/NEJMc2110300 (2021).

11 Abu-Raddad, L. J. *et al.* Effect of mRNA Vaccine Boosters against SARS-CoV-2 Omicron Infection in Qatar. *N Engl J Med* **386**, 1804-1816, doi:10.1056/NEJMoa2200797 (2022).

12 Chemaitelly, H. *et al.* Short- and longer-term all-cause mortality among SARS-CoV-2- infected individuals and the pull-forward phenomenon in Qatar: a national cohort study. *Int J Infect Dis* **136**, 81-90, doi:10.1016/j.ijid.2023.09.005 (2023).

13 AlNuaimi, A. A. *et al.* All-cause and COVID-19 mortality in Qatar during the COVID-19 pandemic. *BMJ Glob Health* **8**, doi:10.1136/bmjgh-2023-012291 (2023).

14 Vogels, C., Fauver, J. & Grubaugh, N. Multiplexed RT-qPCR to screen for SARS-COV-2 B.1.1.7, B.1.351, and P.1 variants of concern V.3. dx.doi.org/10.17504/protocols.io.br9vm966. (2021).

15 Abu-Raddad, L. J., Chemaitelly, H., Butt, A. A. & National Study Group for Covid Vaccination. Effectiveness of the BNT162b2 Covid-19 Vaccine against the B.1.1.7 and B.1.351 Variants. *N Engl J Med* **385**, 187-189, doi:10.1056/NEJMc2104974 (2021).

16 Chemaitelly, H. *et al.* mRNA-1273 COVID-19 vaccine effectiveness against the B.1.1.7 and B.1.351 variants and severe COVID-19 disease in Qatar. *Nat Med* **27**, 1614-1621, doi:10.1038/s41591-021-01446-y (2021).

17 National Project of Surveillance for Variants of Concern and Viral Genome Sequencing. *Qatar viral genome sequencing data. Data on randomly collected samples. https://www.gisaid.org/phylodynamics/global/nextstrain/*, <https://www.gisaid.org/phylodynamics/global/nextstrain/> (2021).

18 Benslimane, F. M. *et al.* One Year of SARS-CoV-2: Genomic Characterization of COVID-19 Outbreak in Qatar. *Front Cell Infect Microbiol* **11**, 768883, doi:10.3389/fcimb.2021.768883 (2021).

19 Hasan, M. R. *et al.* Real-Time SARS-CoV-2 Genotyping by High-Throughput Multiplex PCR Reveals the Epidemiology of the Variants of Concern in Qatar. *Int J Infect Dis* **112**, 52-54, doi:10.1016/j.ijid.2021.09.006 (2021).
20 Saththasivam, J. *et al.* COVID-19 (SARS-CoV-2) outbreak monitoring using wastewater-based epidemiology in Qatar. *Sci Total Environ* **774**, 145608, doi:10.1016/j.scitotenv.2021.145608 (2021).
21 El-Malah, S. S. *et al.* Application of human RNase P normalization for the realistic estimation of SARS-CoV-2 viral load in wastewater: A perspective from Qatar wastewater surveillance. *Environ Technol Innov* **27**, 102775, doi:10.1016/j.eti.2022.102775 (2022).
22 Tang, P. *et al.* BNT162b2 and mRNA-1273 COVID-19 vaccine effectiveness against the SARS-CoV-2 Delta variant in Qatar. *Nat Med* **27**, 2136-2143, doi:10.1038/s41591-021-01583-4 (2021).
23 Chemaitelly, H. *et al.* Duration of mRNA vaccine protection against SARS-CoV-2 Omicron BA.1 and BA.2 subvariants in Qatar. *Nat Commun* **13**, 3082, doi:10.1038/s41467-022-30895-3 (2022).
24 Qassim, S. H. *et al.* Effects of BA.1/BA.2 subvariant, vaccination and prior infection on infectiousness of SARS-CoV-2 omicron infections. *J Travel Med* **29**, doi:10.1093/jtm/taac068 (2022).
25 Altarawneh, H. N. *et al.* Protective Effect of Previous SARS-CoV-2 Infection against Omicron BA.4 and BA.5 Subvariants. *N Engl J Med* **387**, 1620-1622, doi:10.1056/NEJMc2209306 (2022).
26 Chemaitelly, H. *et al.* Protection against Reinfection with the Omicron BA.2.75 Subvariant. *N Engl J Med* **388**, 665-667, doi:10.1056/NEJMc2214114 (2023).
27 World Health Organization (WHO). Living guidance for clinical management of COVID-19. Aavailable from: https://www.who.int/publications/i/item/WHO-2019-nCoV-clinical-2021-2. Accessed on: February 27, 2023. (2021).
28 World Health Organization (WHO). International Guidelines for Certification and Classification (Coding) of COVID-19 as Cause of Death. Available from: https://www.who.int/publications/m/item/international-guidelines-for-certification-and-classification-(coding)-of-covid-19-as-cause-of-death. Accessed on: February 27, 2023. (2020).
