# Supplementary material for: Differential protection against SARS-CoV-2 reinfection pre- and post-Omicron
Source: Nature. 2025 Feb 5;639(8056):1024–31. doi: 10.1038/s41586-024-08511-9 (PMC11946897; doi:10.1038/s41586-024-08511-9)
Supplement: Supplementary file 2 — Reporting Summary [file 41586_2024_8511_MOESM2_ESM.pdf]

nature portfolio

Corresponding author(s): Laith J. Abu-Raddad and Hiam Chemaitelly

Last updated by author(s): Sep 16, 2024

Reporting Summary

Nature Portfolio wishes to improve the reproducibility of the work that we publish. This form provides structure for consistency and transparency in reporting. For further information on Nature Portfolio policies, see our Editorial Policies and the Editorial Policy Checklist.

Statistics

For all statistical analyses, confirm that the following items are present in the figure legend, table legend, main text, or Methods section.

n/a	Confirmed	
☐	☒	The exact sample size (*n*) for each experimental group/condition, given as a discrete number and unit of measurement
☐	☒	A statement on whether measurements were taken from distinct samples or whether the same sample was measured repeatedly
☒	☐	The statistical test(s) used AND whether they are one- or two-sided<br>*Only common tests should be described solely by name; describe more complex techniques in the Methods section.*
☐	☒	A description of all covariates tested
☐	☒	A description of any assumptions or corrections, such as tests of normality and adjustment for multiple comparisons
☐	☒	A full description of the statistical parameters including central tendency (e.g. means) or other basic estimates (e.g. regression coefficient) AND variation (e.g. standard deviation) or associated estimates of uncertainty (e.g. confidence intervals)
☒	☐	For null hypothesis testing, the test statistic (e.g. *F*, *t*, *r*) with confidence intervals, effect sizes, degrees of freedom and *P* value noted<br>*Give P values as exact values whenever suitable.*
☒	☐	For Bayesian analysis, information on the choice of priors and Markov chain Monte Carlo settings
☒	☐	For hierarchical and complex designs, identification of the appropriate level for tests and full reporting of outcomes
☐	☒	Estimates of effect sizes (e.g. Cohen's *d*, Pearson's *r*), indicating how they were calculated

Our web collection on statistics for biologists contains articles on many of the points above.

Software and code

Policy information about availability of computer code

Data collection	Data were entered into the CERNER database system.
Data analysis	Standard epidemiological analyses were conducted using standard commands in STATA/SE 18.0. These commands have been published at: https://github.com/IDEGWCMQ/TestNegCode/blob/main/TestNegCode.txt

For manuscripts utilizing custom algorithms or software that are central to the research but not yet described in published literature, software must be made available to editors and reviewers. We strongly encourage code deposition in a community repository (e.g. GitHub). See the Nature Portfolio guidelines for submitting code & software for further information.

Data

Policy information about availability of data

All manuscripts must include a data availability statement. This statement should provide the following information, where applicable:

- Accession codes, unique identifiers, or web links for publicly available datasets
- A description of any restrictions on data availability
- For clinical datasets or third party data, please ensure that the statement adheres to our policy

The National Coronavirus Disease 2019 (COVID-19) dataset used in this study is a property of the Qatar Ministry of Public Health that was provided to the researchers through a restricted-access agreement that prevents sharing the dataset with a third party or publicly. This dataset encompasses the National COVID-19 Testing Database, the National COVID-19 Vaccination Database, the National COVID-19 Severity Database, and the National Mortality Database. These data are

available under restricted access for preservation of confidentiality of patient data. Access can be obtained through a direct application for data access to Her Excellency the Minister of Public Health (https:// www.moph.gov.qa/english/OurServices/eservices/Pages/Governmental-HealthCommunication-Center.aspx). Data were available to authors through.csv files where information has been downloaded from the CERNER database system (no links/accession codes were available to authors). The raw data are protected and are not available due to data privacy laws. Aggregate data are available within the paper and its supplementary information.

Research involving human participants, their data, or biological material

Policy information about studies with human participants or human data. See also policy information about sex, gender (identity/presentation), and sexual orientation and race, ethnicity and racism.

Reporting on sex and gender	The study populations are balanced by sex (please see Extended Data Table 1). Sex is as recorded in the integrated nationwide digital-health information platform, which is based on the Qatar Identity Card.
Reporting on race, ethnicity, or other socially relevant groupings	The study populations are balanced across nationality groups (please see Extended Data Table 1). Nationality, age, and sex provide a powerful proxy for occupation and socio-economic status in Qatar as evidenced by earlier studies in this population.
Population characteristics	The demographic characteristics of the study populations can be found in Extended Data Table 1.
Recruitment	This is a test-negative case control study where the odds of previous infection was compared between cases (defined as SARS-CoV-2-positive tests) and controls (defined as SARS-CoV-2-negative tests) before and after the the introduction of the omicron variant in Qatar on December 19, 2021. Two analyses were conducted. The first analysis was conducted between February 5, 2020 (the onset of the COVID-19 pandemic in Qatar), and December 18, 2021 to assess the effectiveness of a pre-omicron infection in preventing reinfection with a pre-omicron virus. The second analysis was conducted between December 19, 2021, and February 12, 2024 (marking the end of the study) to assess the effectiveness of an omicron infection in preventing reinfection with an omicron virus. COVID-19 laboratory testing, clinical infection data, severity, hospitalization, vaccination, and related demographic details were extracted from the integrated nationwide digital-health information platform that hosts the national, federated SARS-CoV-2 databases. These databases are complete with no missing information for PCR testing, medically-supervised rapid antigen testing, COVID-19 vaccinations, COVID-19 hospitalizations and deaths, and basic demographic details, and have captured all SARS-CoV-2-related data since epidemic onset. This study was conducted on the entire resident population of Qatar. SARS-CoV-2 testing was extensive in Qatar until October 31, 2022, with nearly 5% of the population being tested every week, primarily for routine purposes such as screening or meeting travel-related requirements. Subsequently, testing rates decreased, with less than 1% of the population being tested per week. The majority of infections during the pandemic were diagnosed through routine testing rather than symptomatic presentation.
Ethics oversight	The study was approved by the Hamad Medical Corporation and Weill Cornell Medicine-Qatar Institutional Review Boards with waiver of informed consent.

Note that full information on the approval of the study protocol must also be provided in the manuscript.

Field-specific reporting

Please select the one below that is the best fit for your research. If you are not sure, read the appropriate sections before making your selection.

☒ Life sciences ☐ Behavioural & social sciences ☐ Ecological, evolutionary & environmental sciences

For a reference copy of the document with all sections, see nature.com/documents/nr-reporting-summary-flat.pdf

Life sciences study design

All studies must disclose on these points even when the disclosure is negative.

Sample size	COVID-19 laboratory testing, clinical infection data, severity, hospitalization, vaccination, and related demographic details were extracted from the integrated nationwide digital-health information platform that hosts the national, federated SARS-CoV-2 databases. These databases are complete and have captured all SARS-CoV-2-related data since epidemic onset. The data is for the entire national population and includes every individual tested for SARS-CoV-2 in Qatar. The sample size varied depending on the definition used for cases (SARS-CoV-2-positive tests, as well as severe, critical, or fatal COVID-19 due to a SARS-CoV-2 infection), and controls (SARS-CoV-2-negative tests), during each of the pre-omicron and omicron phases of the pandemic. Cases and controls were matched exactly one-to-two by sex, 10-year age group, nationality, number of coexisting conditions, number of vaccine doses, calendar week of the SARS-CoV-2 test, method of testing (PCR or rapid antigen), and reason for testing in estimating the effectiveness of previous infection in preventing reinfection. A one-to-five matching ratio was applied in estimating effectiveness in preventing severe, critical, or fatal COVID-19 upon reinfection, to enhance statistical precision.<br>In each analysis for a specific time-since-previous-infection stratum, we included only those with previous infection in this specific time-since-previous-infection stratum and those with no previous infection (our reference group). Thus, the number of cases (and controls) varied across time-since-previous-infection analyses. Given that the sample sizes were based on the entire national population with only individuals that do not fit the eligibility criteria excluded, the sample size for each sub-study can be considered sufficient. Detailed sample sizes can be found in Extended Data Figs. 1 and 2, Extended Data Table 1, and Table 1.
Data exclusions	Exclusion criteria were specified a priori. SARS-CoV-2 reinfection is conventionally defined as a documented infection ≥90 days after a previous infection, to avoid misclassifying prolonged test positivity as a reinfection with shorter time intervals. Consequently, cases or controls preceded by SARS-CoV-2-positive tests within 90 days were excluded. To comply with the non-differential healthcare-seeking behavior assumption inherent to the test-negative study design, only tests with a documented reason for testing were included in the analysis. In the

	omicron-era analysis, cases or controls preceded by a pre-omicron infection were excluded from the analysis, as the research question pertained to the effectiveness of omicron immunity, rather than pre-omicron immunity, against omicron reinfection.
Replication	To validate the results and ensure reproducibility, six additional independent analyses were conducted to estimate the effectiveness of previous infection in preventing reinfection overall and by time since previous infection after 1) restricting the analysis to tests performed due to clinical suspicion, indicating the presence of symptoms consistent with a respiratory tract infection, 2) stratifying the analysis by vaccination status, 3) redefining SARS-CoV-2 reinfection as a documented infection occurring ≥40 days after a previous infection, instead of the conventional ≥90 days, 4) removing the requirement of matching by the number of coexisting conditions, 5) using a cohort study design to estimate natural infection protection instead of a test-negative design, and 6) using mathematical modeling to evaluate the impact of misclassification of prior infection status on the estimated waning pattern of immune protection. All analyses confirmed/reproduced estimates of the effectiveness of previous infection against reinfection obtained in the main analysis.
Randomization	Not applicable as this is an observational case-control study where individuals are aware of their infection status. However, controls were selected from the entire national population, and exact matching on multiple factors was employed to ensure rigorous pairing of cases and controls. To further ensure that effectiveness estimates were not biased, the study design controlled for vaccination status, allowing for differentiation between the effects of prior infection and vaccination, another strength of the test-negative design.
Blinding	Not applicable as this is an observational case-control study where individuals are aware of their infection status.

Reporting for specific materials, systems and methods

We require information from authors about some types of materials, experimental systems and methods used in many studies. Here, indicate whether each material, system or method listed is relevant to your study. If you are not sure if a list item applies to your research, read the appropriate section before selecting a response.

Materials & experimental systems

n/a	Involved in the study
☒	☐ Antibodies
☒	☐ Eukaryotic cell lines
☒	☐ Palaeontology and archaeology
☒	☐ Animals and other organisms
☒	☐ Clinical data
☒	☐ Dual use research of concern
☒	☐ Plants

Methods

n/a	Involved in the study
☒	☐ ChIP-seq
☒	☐ Flow cytometry
☒	☐ MRI-based neuroimaging
